# Cell-autonomous retinoic acid receptor signaling has stage-specific effects on mouse enteric nervous system

**DOI:** 10.1172/jci.insight.145854

**Published:** 2021-05-24

**Authors:** Tao Gao, Elizabeth C. Wright-Jin, Rajarshi Sengupta, Jessica B. Anderson, Robert O. Heuckeroth

**Affiliations:** 1 Children’s Hospital of Philadelphia Research Institute, Philadelphia, Pennsylvania, USA.; 2 Department of Pediatrics, Washington University School of Medicine, St. Louis, Missouri, USA.; 3 Department of Pediatrics, Perelman School of Medicine at the University of Pennsylvania, Philadelphia, Pennsylvania, USA.

**Keywords:** Gastroenterology, Embryonic development, Neurodevelopment

## Abstract

Retinoic acid (RA) signaling is essential for enteric nervous system (ENS) development, since vitamin A deficiency or mutations in RA signaling profoundly reduce bowel colonization by ENS precursors. These RA effects could occur because of RA activity within the ENS lineage or via RA activity in other cell types. To define cell-autonomous roles for retinoid signaling within the ENS lineage at distinct developmental time points, we activated a potent floxed dominant-negative RA receptor α (RarαDN) in the ENS using diverse CRE recombinase–expressing mouse lines. This strategy enabled us to block RA signaling at premigratory, migratory, and postmigratory stages for ENS precursors. We found that cell-autonomous loss of RA receptor (RAR) signaling dramatically affected ENS development. CRE activation of RarαDN expression at premigratory or migratory stages caused severe intestinal aganglionosis, but at later stages, RarαDN induced a broad range of phenotypes including hypoganglionosis, submucosal plexus loss, and abnormal neural differentiation. RNA sequencing highlighted distinct RA-regulated gene sets at different developmental stages. These studies show complicated context-dependent RA-mediated regulation of ENS development.

## Introduction

The enteric nervous system (ENS) is a complex network of neurons and glia that resides in the bowel wall and is essential for intestinal function (1, 2). These ENS cells arise primarily from vagal enteric neural crest-derived cell (ENCDC) precursors that divide rapidly and colonize the bowel in a rostral to caudal direction from E9 to E13.5 in mice (3–6). In addition to vagal ENCDC, ENS precursors include sacral neural crest (7), mesenteric neural crest (8), sympatho-enteric precursors (9), Schwann cells (10), and perhaps bowel epithelial cells (11). As ENCDC colonize the bowel, they differentiate into about 20 neuron types, and many types of glia that form extensive networks to control most aspects of bowel function (2, 12–17). Maturation of the ENS continues during fetal development, and remodeling continues after birth (18–20). Retinoic acid (RA), the active metabolite of vitamin A, is an important morphogen with an integral role in ENCDC migration, proliferation, and differentiation (21–32). RA functions mainly as a transcriptional regulator that binds to and activates RA receptor/retinoid x receptor (RAR/RXR) heterodimers (33). RAR/RXR heterodimers bind DNA at RA response elements (RAREs) to regulate transcriptional activity. Several studies demonstrate vital roles for vitamin A and its active metabolite RA in ENS development. In rat and mouse primary cell culture, RA increases ENCDC proliferation and neuronal differentiation while decreasing neurite length (23, 27). In vivo, vitamin A–deficient mice (*Rbp4^–/–^* mice on a vitamin A–deficient diet) develop distal bowel aganglionosis (26), confirming that vitamin A is needed for bowel colonization by ENCDC. The extent of bowel aganglionosis dramatically increased when *Ret* heterozygosity was combined with vitamin A deficiency, suggesting potent gene-environment interactions in mice (26). Consistent with these observations, loss of the primary RA synthesis enzyme retinaldehyde dehydrogenase 2 (*Raldh2*) also causes severe intestinal aganglionosis in mice (29), with more minor effects of murine *Raldh1* and *Raldh3* mutations (25). Furthermore, maternal vitamin A deficiency or excess can cause intestinal hypoganglionosis without aganglionosis in rats and mice (22, 28, 32). While these results clearly show that RA signaling is needed for ENS development, many questions remain. First, because RAR and RXR receptors are expressed in diverse cell types (27), it is not clear whether RA acts directly on ENCDC or via effects on neighboring cells. Second, effects of RA signaling at distinct developmental stages remain elusive. Third, the RA-targeted gene network in ENCDC in vivo is not yet established. To address these questions, we employed a very potent CRE-dependent dominant-negative RA receptor α (*RarαDN^Loxp–STOP–LoxP/+^*; RAR*α*DN) (34) in combination with fluorescence-based lineage tracing in mice. We discovered that RA signaling regulates ENS development in a cell-autonomous manner, with distinct effects on different developmental stages. Furthermore, gene expression profiling showed stage-specific effects of blocking RAR signaling in developing ENCDC. These results suggest that vitamin A deficiency or excess could alter ENS structure and function in many ways during intrauterine and postnatal periods, contributing to human bowel motility disorders.

## Results

### Cell-autonomous RAR signaling in neural crest derivatives is required for craniofacial and ocular development.

**Figure 1:**
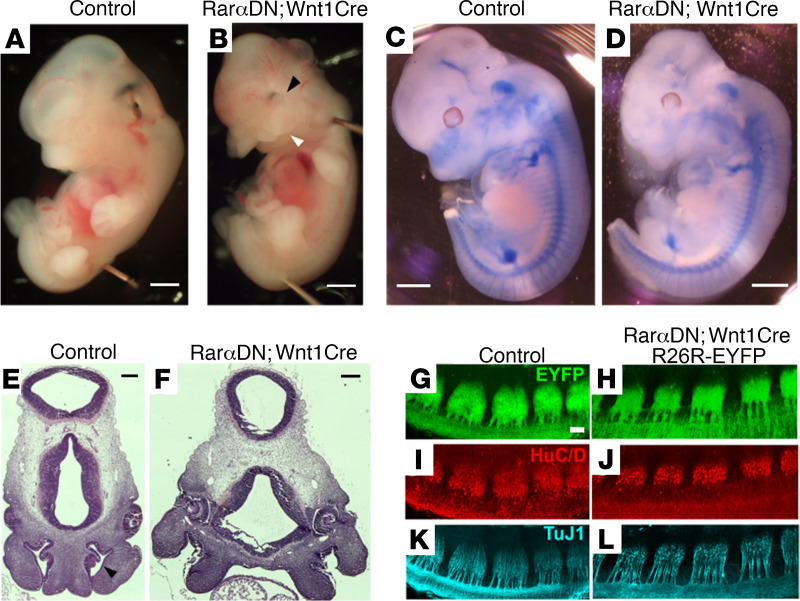
*RarαDN^LoxP/+^; Wnt1Cre^+^* mice have abnormal craniofacial development. (**A** and **B**) E12.5 *RarαDN^LoxP/+;^ Wnt1Cre^+^* (*RarαDN; Wnt1Cre*) mice have obvious craniofacial defects with inset eyes (black arrowhead) and midline facial cleft (white arrowhead). Scale bar: 1 mm. *n =* 3 control, *n =* 3 *RarαDN^LoxP/+^; Wnt1Cre^+^*. (**C** and **D**) E12.5 Alcian blue staining shows markedly reduced cartilage in neural crest–derived structures of the face. Scale bar: 1mm. *n =* 3 control, *n =* 3 *RarαDN^LoxP/+^; Wnt1Cre^+^*. (**E** and **F**) Representative images of H&E-stained coronal sections of *RarαDN^LoxP/+^; Wnt1Cre^+^* mice show inset eyes and cleft nasal passages compared with control (*Wnt1Cre^+^*) at E12.5. Arrowhead indicates nasal cavity. Scale bar: 200 μm. *n =* 3 control, *n =* 3 *RarαDN^LoxP/+^; Wnt1Cre^+^*. (**G**–**J**) Dorsal root ganglia appear fairly normal at E11.5 in *RarαDN^LoxP/+^; Wnt1Cre^+^; R26R-EYFP^+^* mice based on EYFP (**G** and **H**), HuC/D^+^ neuron (**I** and **J**), and TuJ1^+^ neurite imaging (**K** and **L**). Scale bar: 100 μm. *n =* 3 control, *n =* 3 *RarαDN^LoxP/+^; Wnt1Cre^+^*.

To characterize cell-autonomous roles for RAR signaling within the ENS lineage, we bred *RarαDN^LoxP/+^* to *Wnt1Cre^+^* mice. *RarαDN^LoxP/+^* produce RAR*α*DN after CRE-mediated DNA recombination. *Wnt1Cre* express *Cre* recombinase in the CNS and many neural crest derivatives, including essentially all fetal ENS precursors (35–37). *RarαDN^LoxP/+^; Wnt1Cre*^+^ mice are viable at E12.5, but they die by E14.5 (Table 1). E12.5 *RarαDN^LoxP/+^; Wnt1Cre*^+^ have major malformations of neural crest–derived facial structures (Figure 1, A and B) with absent facial cartilage (Figure 1, C and D). Sectioning showed failure of nasomedial process fusion at the midline and a wide frontonasal region (Figure 1, E and F). In contrast, dorsal root ganglia (DRG), another crest-derived structure, appeared fairly normal even though *Wnt1Cre* induced recombination of an EYFP reporter in DRG (Figure 1, G–L). These data highlight distinct RA roles in different neural crest–derived tissues during development. Our primary goal was to investigate RA signaling effects in the ENS.

### ENS development requires cell autonomous RAR signaling.

**Figure 2:**
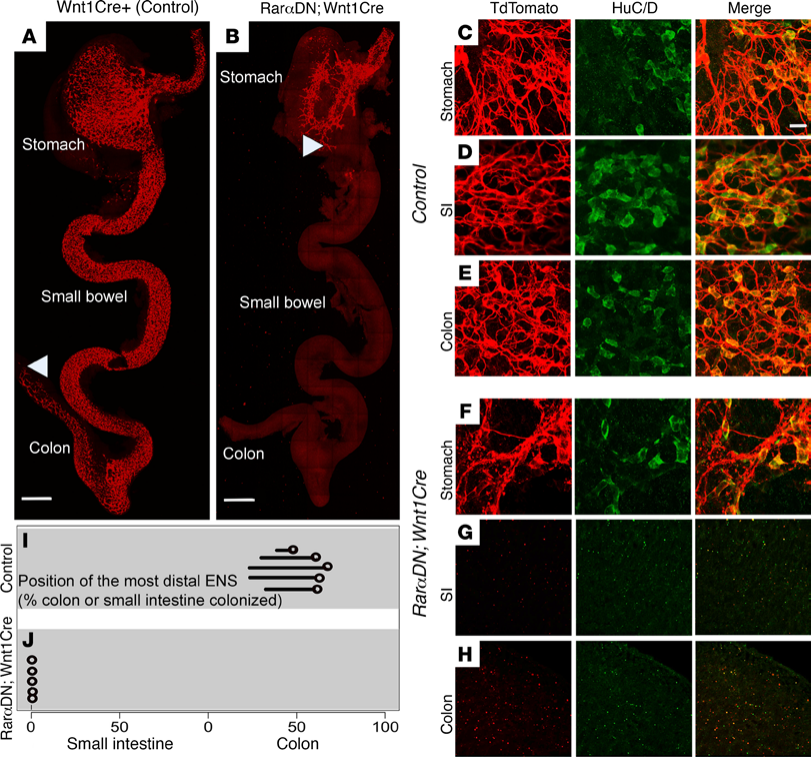
Cell-autonomous RAR signaling is required for ENS precursor colonization of fetal small bowel and colon. (**A** and **B**) Representative images of bowel colonization by TuJ1^+^ enteric neurons (red) at E12.5. (**A**) *Wnt1Cre^+^* controls have TuJ1^+^ cells throughout the bowel from esophagus to midcolon. (**B**) *RarαDN^LoxP/+^; Wnt1Cre^+^* mice only have TuJ1^+^ cells present in the esophagus and stomach. A white arrowhead shows the position of the most distal TuJ1^+^ cell or neurite in each image. Scale bars: 500 μm. (**C**–**H**) Representative E12.5 images of stomach, small intestine (SI), and colon show cells stained with TdTomato^+^ (red) and HuC/D (green) throughout the bowel in control (**C**–**E**), whereas TdTomato^+^- and HuC/D-stained cells are absent in SI and colon of *RarαDN^LoxP/+^; Wnt1Cre^+^* mice (**F**–**H**). (**C**–**H**) Scale bar: 150 μm. (**I** and **J**) Circles show the position of the most distal TdTomato^+^ ENS cell in control or *RarαDN^LoxP/+^; Wnt1Cre^+^* mice at E12.5. The line attached to each circle indicates a hypoganglionic zone in controls. *n =* 5 controls, *n =* 5 *RarαDN^LoxP/+^; Wnt1Cre^+^*.

At E12.5 in *Wnt1Cre^+^* (control) mice, ENCDC had colonized the esophagus, stomach, small intestine, and half of the colon, as seen by TuJ1 (neuron specific β3-tubulin) antibody staining (Figure 2A). In contrast, E12.5 *RarαDN^LoxP/+^; Wnt1Cre^+^* mice only had TuJ1^+^ cells in the esophagus and stomach (Figure 2B). Recognizing that the absence of TuJ1 staining might reflect impaired neuronal differentiation in *RarαDN^LoxP/+^; Wnt1Cre^+^* mice or could reflect absent ENS, we bred to *R26R-TdTomato* lineage reporter mice so that cells undergoing CRE-mediated DNA recombination are unambiguously marked. While TdTomato^+^ ENS and HuC/D^+^ (neuronal RNA binding protein) neurons were readily detected in stomach, small bowel, and proximal colon of control animals (Figure 2, C–E and I), the *RarαDN^LoxP/+^; Wnt1Cre^+^; R26R-TdTomato^+^* mice did not have TdTomato^+^ or HuC/D^+^ cells in the small bowel or colon and had fewer ENS cells in stomach than controls (Figure 2, F–H and J). These analyses confirmed that blocking cell-autonomous RAR signaling in the *Wnt1Cre* lineage completely prevented these ENS precursors from colonizing the small bowel and colon.

### Blocking RAR signaling causes defects in ENCDC migration and differentiation by E10.5.

**Figure 3:**
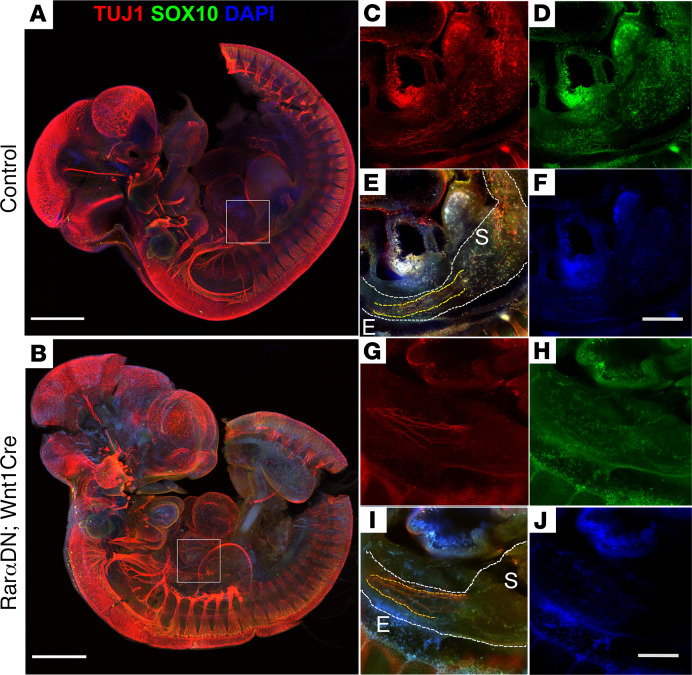
Blocking RAR signaling impairs ENCDC migration and differentiation at E10.5. (A and B) Whole embryo imaging showing E10.5 *RarαDN^LoxP/+^* (control) (A) or E10.5 *RarαDN^LoxP/+^; Wnt1Cre^+^* (B) mice stained with TUJ1 (red) and SOX10 (green) antibodies plus DAPI (blue). Scale bar: 500 μm. (**C**–**J**) Zoomed-in images of selected slices from the *Z*-stack of the whole embryos show esophagus and stomach from control (**C**–**F**) or *RarαDN^LoxP/+^; Wnt1Cre^+^* (**G**–**J**) mice. Scale bar: 100 μm. In control embryos, there are readily visible SOX10^+^TUJ1^+^ cells in both esophagus and stomach area (**C**–**F**). In contrast, there are almost no SOX10^+^ cells in the mutant stomach, even though SOX10^+^ cells were observed in the esophagus (**G**–**J**). (**E** and **I**) White dotted lines outline of esophagus and stomach. Yellow dotted lines highlight the region with extrinsic vagal nerve fibers. E, esophagus; S, stomach. These are representative images from *n =* 3 *RarαDN^LoxP/+^* (control), *n =* 3 *RarαDN^LoxP/+^; Wnt1Cre^+^*.

To determine if *RarαDN* expression within neural crest–derived ENS precursors acts at earlier developmental stages, we examined E10.5 whole embryo via 3DISCO tissue clearing (38) and confocal microscopy (Figure 3, A and B). TuJ1 and SOX10 antibody staining showed many ENCDC in the esophagus and stomach in control mice (Figure 3, C–F) but very few stained ENCDC in the proximal bowel of *RarαDN^LoxP/+^; Wnt1Cre^+^* mice (Figure 3, G–J), consistent with a defect in early stages of bowel colonization. More specifically, while there were some SOX10^+^ ENCDC near the vagus in mutant mice (Figure 3, G–J), the control mice had many SOX10^+^ ENCDC that had migrated far beyond the vagus and into the stomach (Figure 3, C–F). Furthermore, in control mice, many ENCDC were TuJ1 immunoreactive, suggesting early neuronal differentiation (Figure 3, C and E), but there were almost no TuJ1^+^ cells in the esophagus or stomach of *RarαDN^LoxP/+^; Wnt1Cre^+^* mice (Figure 3, G and I). Collectively, these data suggest that cell-autonomous RAR signaling is needed for ENCDC to populate the bowel and differentiate into neurons.

### Cell autonomous RAR signaling is required for RET and PHOX2B expression in ENCDC.

**Figure 4:**
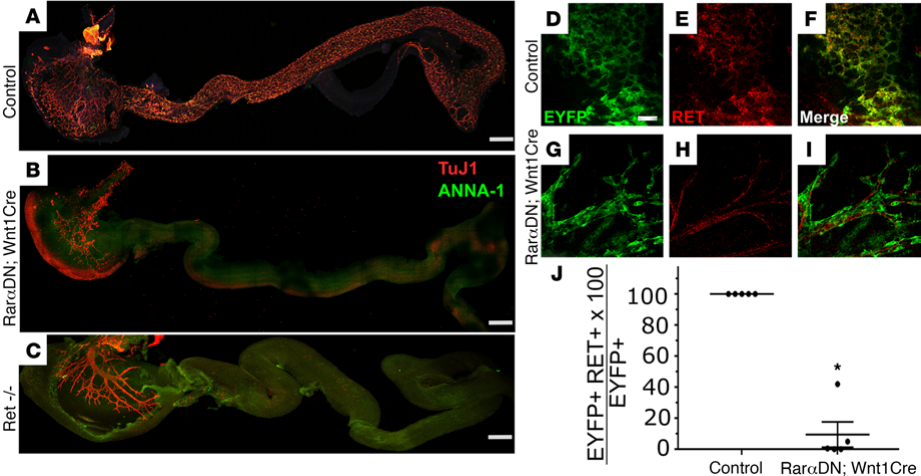
RAR signaling in ENCDC is required for Ret expression. (**A**–**C**) Whole-mount TuJ1 and HuC/D antibody staining of E12.5 bowel from control, *RarαDN^LoxP/+^; Wnt1Cre^+^*, *and Ret^–/–^* mice. Note that there are no ENCDC beyond stomach in *RarαDN^LoxP/+^; Wnt1Cre^+^* (**B**) or *Ret^–/–^* mice (**C**). Scale bar: 1000 μm. (**D**–**I**) IHC of E12.5 control (*Wnt1Cre^+^; Rosa26^EYFP^*) (**D**, **E**, and **F**) or *RarαDN^LoxP/+^; Wnt1Cre^+^; Rosa26^EYFP^* (**G**, **H**, and **I**) stomach using EYFP and RET antibodies. Scale bar: 50 μm. (**J**) Percentage ratio of EYFP^+^ ENCDC that are RET immunoreactive. *Wnt1Cre^+^; Rosa26^EYFP^* (*n =* 5 mice, 2100 cells), *RarαDN^LoxP/+^; Wnt1Cre^+^; Rosa26^EYFP^* (*n =* 5 mice, 1994 cells, *P =* 0.0002, 2-tailed unpaired Student’s *t* test).

**Figure 5:**
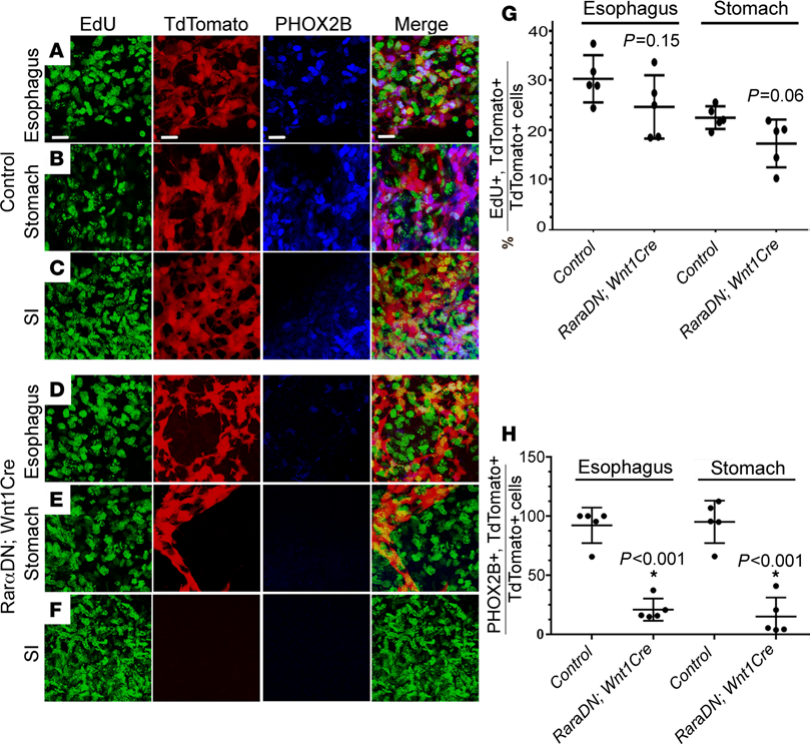
RAR signaling in ENCDC is required for PHOX2B expression. (**A**–**F**) *Wnt1Cre^+^; R26R-TdTomato* (control) and *RarαDN^LoxP/+^*; *Wnt1Cre^+^*; *R26R-TdTomato* E12.5 bowel whole-mount showing EdU and PHOX2B IHC, as well as TdTomato fluorescence in esophagus (**A** and **D**), stomach (**B** and **E**), and small intestines (**C** and **F**). (**G**) Cell proliferation rate (EdU^+^TdTomato^+^/TdTomato^+^ × 100) (*n =* 5 mice, 718 cells) was similar in *Wnt1Cre^+^; R26R-TdTomato* (control) and *RarαDN^LoxP/+^; Wnt1Cre^+^* mice*; R26R-TdTomato* (*n =* 5 mice, 643 cells). (**H**) Quantitative analysis of PHOX2B expression in ENCDC (PHOX2B^+^TdTomato^+^/TdTomato^+^ × 100) showed few mutant ENCDC are PHOX2B^+^. *Wnt1Cre^+^; R26R-TdTomato* (control) (*n =* 5 mice, 718 cells) and *RarαDN^LoxP/+^; Wnt1Cre^+^; R26R-TdTomato* (*n =* 5 mice, 643 cells). Scale bar: 20 μm. Two-tailed unpaired Student’s *t* tests were used for statistics.

The defect in bowel colonization by ENCDC of *RarαDN^LoxP/+^; Wnt1Cre^+^* mice closely resembles the phenotype in *Ret* and *Phox2b* null mice (39, 40) (Figure 4, A–C). Because RA was previously reported to induce *Ret* expression in quail ENCDC migrating from the neural tube to bowel (24), we stained E12.5 bowel from *RarαDN^LoxP/+^; Wnt1Cre^+^; R26R-EYFP^+^* and controls (*Wnt1Cre^+^; R26R-EYFP^+^*) with RET antibody. A total of 100% of stomach EYFP^+^ ENCDC expressed RET in controls as expected (Figure 4, D–F). Curiously, while 1 *RarαDN^LoxP/+^; Wnt1Cre^+^; EYFP^+^* mouse had many RET^+^EYFP^+^ ENCDC in the stomach (42%), the other mice evaluated had very few RET^+^EYFP^+^ ENCDC (0%, 0%, 0.4%, and 4.9%) (Figure 4, G–J). RAR*α*DN also dramatically reduced the percentage of lineage-marked ENCDC that express PHOX2B in the stomach or esophagus of *RarαDN^LoxP/+^; Wnt1Cre^+^; TdTomato* mice (Figure 5, A–F and H). EdU incorporation into lineage marked ENCDC in E12.5 stomach of *RarαDN^LoxP/+^; Wnt1Cre^+^; TdTomato* mice, however, was not statistically different from controls (Figure 5, A–F and G); therefore, it is not clear that reduced proliferation within stomach ENCDC fully accounts for the phenotype. Collectively, these data suggest that RAR signaling is needed in the *Wnt1Cre* ENS lineage for *Ret* and *Phox2b* expression, further suggesting that loss of either RET or PHOX2B could cause this type of extensive intestinal aganglionosis (39, 40).

### Loss of RAR signaling causes defective vagal nerve development.

**Figure 6:**
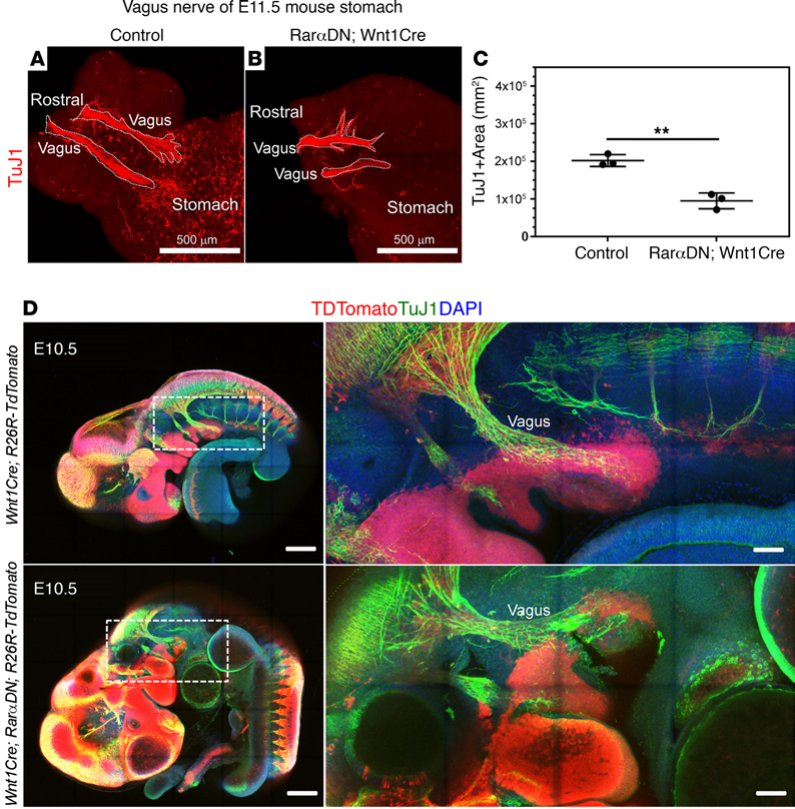
Vagal nerve fibers occupy a smaller area in E11.5 stomach of *RarαDN^LoxP/+^; Wnt1Cre^+^* mice than in controls. (**A** and **B**) TuJ1 antibody–stained E11.5 stomach of *RarαDN^LoxP/+^* (control) (**A**) or *RarαDN^LoxP/+^; Wnt1Cre^+^* (**B**) mice. Scale bar: 500 μm. (**C**) Quantification of TuJ1^+^ stained vagal fiber area. **P < 0.01 by 2 tailed unpaired Student’s *t* tests. (**D**) Whole embryo imaging of E10.5 *Wnt1Cre^+^; R26R-TdTomato^+^* and *RarαDN^LoxP/+^; Wnt1Cre^+^; R26R-TdTomato^+^*. Scale bars: 500 μm (left) and 200 μm (right, enlarged). Note that TuJ1^+^ vagal nerve fibers are not TdTomato^+^. Box shows region of the magnified image. *n =* 3, *Wnt1Cre^+^; R26R-TdTomato^+^*. *n =* 3, *RarαDN^LoxP/+^; Wnt1Cre^+^; R26R-TdTomato^+^*. Supplemental Video 1 and Supplemental Video 2 show 3-dimensional *Z*-stacks from embryos in **D**.

In parallel with the loss of ENCDC in fetal stomach, E11.5 *RarαDN^LoxP/+^; Wnt1Cre^+^* mice had smaller vagus nerves than control littermates (Figure 6, A–C). The reduction in vagal nerve fibers in the stomach appears to be non–cell autonomous because TuJ1^+^ vagal nerve fibers are TdTomato-negative in *Wnt1Cre^+^; TdTomato^+^* and *RarαDN^+^; Wnt1Cre^+^; TdTomato^+^* mice (Figure 6D, Supplemental Figure 1, and Supplemental Videos 1 and 2; supplemental material available online with this article; https://doi.org/10.1172/jci.insight.145854DS1). Enlarged images also demonstrate many TdTomato^+^ cells migrating along the TuJ1^+^ vagal nerve fibers in *Wnt1Cre^+^; TdTomato* and *RarαDN; Wnt1Cre^+^; TdTomato* mice (Supplemental Figure 1, E, F, K, and L). Furthermore, while ENCDC migrate far beyond the vagus in control animals, TuJ1^+^- and SOX10-labeled ENCDC remained close to vagal fibers in E11.5 *RarαDN^LoxP/+^; Wnt1Cre^+^* mice (Figure 3, D and H, and Figure 6, A and B). These observations suggest synergistic interactions between growing vagal nerve fibers and migrating ENCDC that colonize the bowel to form ENS.

### Loss of RAR signaling in SOX10 lineage of ENCDC causes aganglionosis.

**Figure 7:**
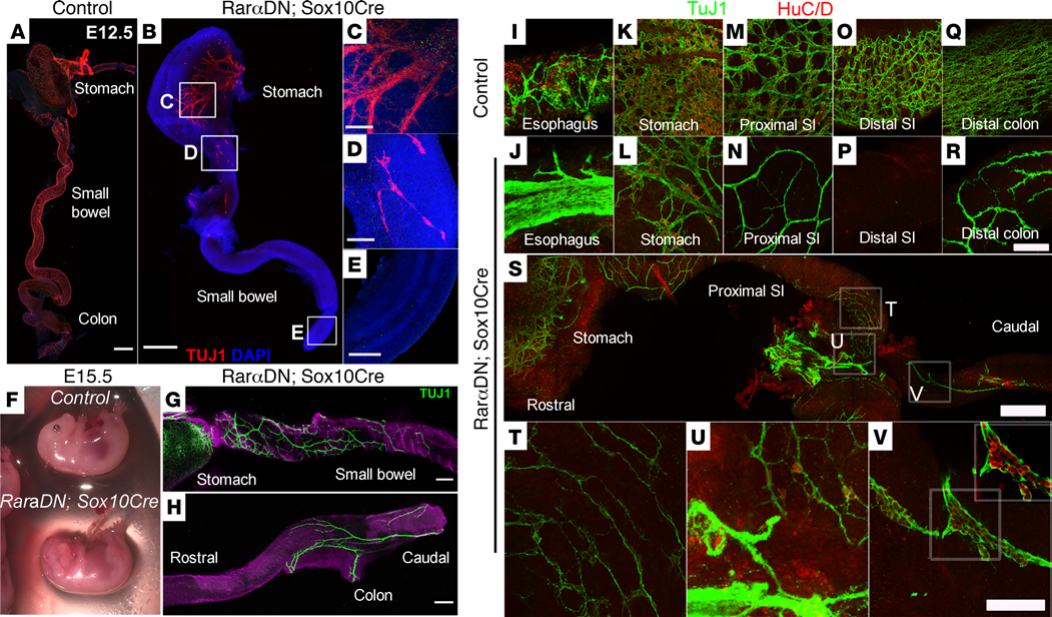
*RarαDN^LoxP/+^; Sox10Cre^+^* mice had extensive distal bowel aganglionosis. (**A**–**E**) E12.5 control or *RarαDN^LoxP/+^; Sox10Cre^+^* whole bowel was stained with TuJ1 antibody (red) and DAPI (blue). Insets highlight mutant stomach (**C**), proximal small intestine (**D**), and distal small intestine (**E**). Sparse TuJ1^+^ nerve cell bodies were seen in the proximal small bowel (**D**) but not in more distal small bowel (**E**) of *RarαDN^LoxP/+^; Sox10Cre^+^* mice. Scale bars: 500 μm (**A** and **B**) and 100 μm (**C**–**E**). (**F**) Images of either *RarαDN^LoxP/+^* (control) or *RarαDN^LoxP/+^; Sox10Cre^+^* E15.5 embryos. Note the defective eye and craniofacial development of the mutant embryos. (**G** and **H**) E15.5 *RarαDN^LoxP/+^; Sox10Cre^+^* bowels were stained with TuJ1 (green) and counter-stained with DAPI. Note the TuJ1^+^ network at proximal small intestine (**G**) and distal colon (**H**). Scale bars: 100 μm. (**I**–**R**) *RarαDN^LoxP/+^* (control) or *RarαDN^LoxP/+^; Sox10Cre^+^* bowels were stained with HuC/D (red) and TuJ1 (green) antibodies. Representative images of esophagus (**I** and **J**), stomach (**K** and **L**), proximal small intestine (**M** and **N**), distal small intestine (**O** and **P**), and end of distal colon (**Q** and **R**). Scale bar: 50 μm. (**S**) Proximal small intestine of E15.5 *RarαDN^LoxP/+^; Sox10Cre^+^* bowel. Regions outlined by boxes indicate which areas are enlarged in (**T**–**V**). Note extrinsic nerve fibers (**T** and **U**). Scale bars: 500 μm (**S**) and 50 μm (**T**–**V**). *n =* 3, *RarαDN^LoxP/+^* (control); *n =* 3, *RarαDN^LoxP/+^; Sox10Cre^+^*.

To evaluate cell-autonomous effects of RAR beyond E12.5 when *RarαDN^LoxP/+^; Wnt1Cre^+^* die, we generated *RarαDN^LoxP/+^; SOX10Cre^+^* mice. These mice express *Cre* from *Sox10* regulatory elements, beginning at E8.5 when ENCDC migrate through the paraxial mesoderm to foregut (41). To confirm CRE activation in the ENS lineage, we examined *SOX10Cre; R26R-TdTomato* mice at E12.5 and found essentially complete overlap of the TdTomato lineage marker and RET antibody staining in the bowel (Supplemental Figure 2). Unlike controls (Figure 7A) and like *RarαDN^LoxP/+^; Wnt1Cre^+^*, the E12.5 *RarαDN^LoxP/+^; SOX10Cre^+^* had TuJ1^+^ ENCDC predominantly confined to the stomach at E12.5 (Figure 7, B–E), with a few sparsely distributed TuJ1^+^ ENCDC in the proximal small intestine (Figure 7D). By E15.5, *RarαDN^LoxP/+^; SOX10Cre^+^* had obvious eye and craniofacial defects but remained viable (Figure 7F). The E15.5 ENS network was dense in the esophagus and stomach but was sparse in the proximal small intestine (Figure 7, G, J, L, and N) compared with controls (Figure 7, I, K, and M). Distal small intestine (Figure 7P) and colon were aganglionic with extrinsic nerve fibers in the distal colon (Figure 7, H and R) of mutant mice in contrast to controls (Figure 7, O and Q). Neurons in the small bowel were often associated with extrinsic nerve fibers and separated from more proximal ENS (Figure 7, S–V). These data are consistent with the hypothesis that RAR signaling is required as ENCDC migrate from the neural tube to bowel and that it is not needed by ENS precursors before E8.5 in the neural tube.

### Blocking RAR signaling in the TyrCre lineage reduces enteric neurons and alters ENS patterning.

**Figure 8:**
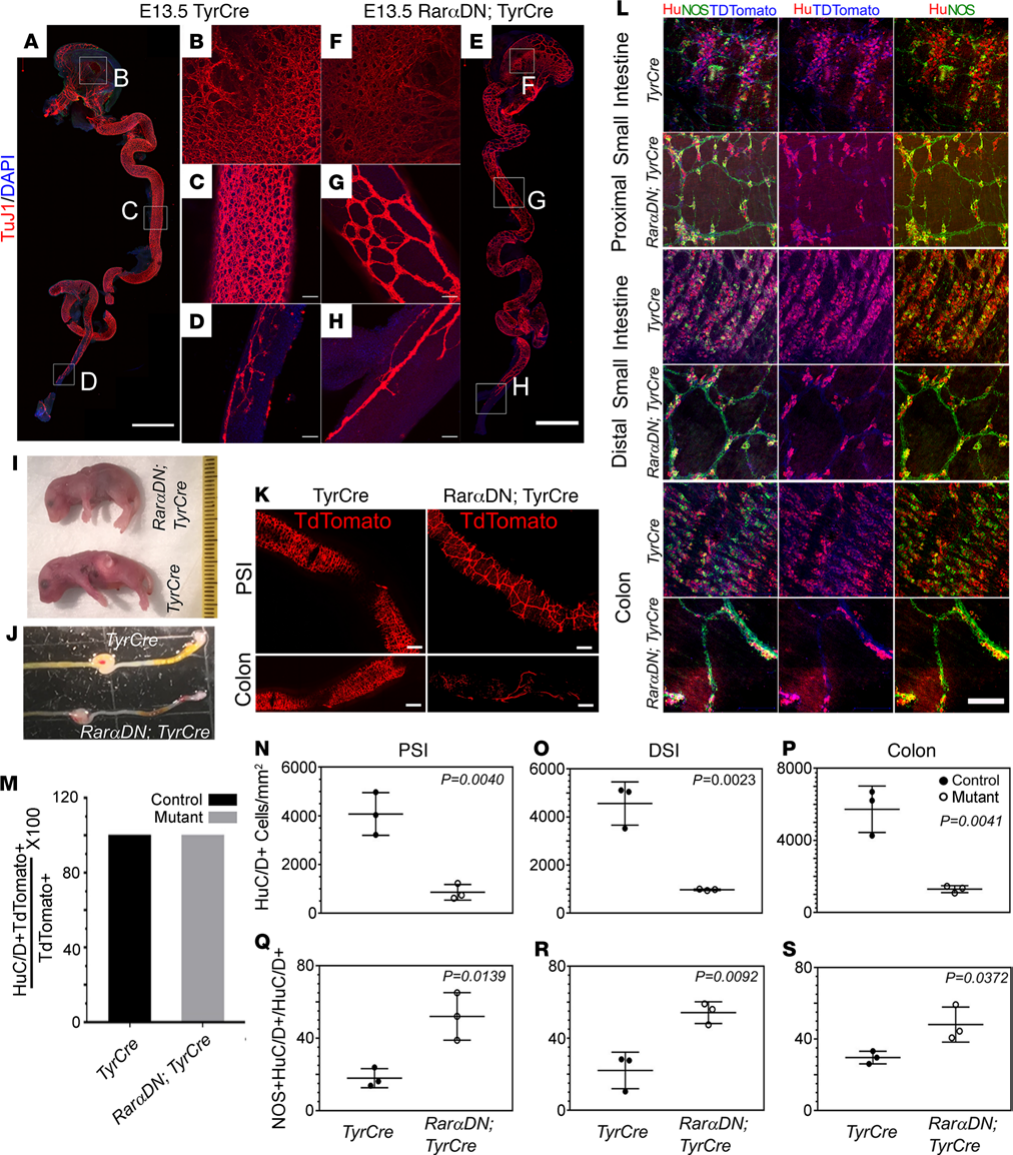
TyrCre-driven *RarαDN* reduces enteric neuron number and causes abnormal ENS patterning. (**A**–**H**) E13.5 *RarαDN^LoxP/+^; TyrCre^+^* or *TyrCre^+^* (control) bowels stained with TuJ1 antibody (red) and DAPI. Scale bars: 1 mm (**A** and **E**). Boxes in **A** and **E** indicate regions of stomach (**B** and **F**), midgut (**C** and **G**), and distal colon (**D** and **H**) that are magnified in adjacent images. Scale bars: 50 μm (**B**–**H**). (**I**) *RarαDN^LoxP/+^; TyrCre^+^* appear similar to control (*TyrCre^+^*) at birth but did not feed and lack a milk spot in stomach (*n =* 6). (**J**) *RarαDN^LoxP/+^; TyrCre^+^* distal bowel appeared grossly similar to control (*TyrCre^+^*) at P0 (*n =* 10). (**K**) TdTomato^+^ images of proximal small intestine (PSI) and colon of either *RarαDN^LoxP/+^; TyrCre^+^* or *TyrCre^+^* (control) E18.5 pups. Scale bar: 1 mm. (**L**) Whole-mount IHC of E18.5 *RarαDN^LoxP/+^; TyrCre^+^* or *TyrCre^+^* (control) bowels using HuC/D (red) and NOS1 (green) antibodies and via TdTomato (blue) fluorescence. Scale bar: 100 μm. (**M**) All TdTomato^+^ cells in the small bowel and colon were HuC/D^+^ in E18.5 *RarαDN^LoxP/+^; TyrCre^+^* and *TyrCre^+^* (control) mice. (**N**–**S**) Total HuC/D^+^ neurons were reduced in PSI, distal small intestine (DSI) and colon of *RarαDN^LoxP/+^; TyrCre^+^* mice (**N**–**P**), but the percentage of HuC/D^+^ neurons expressing NOS1 was increased in mutants (**Q**–**S**). *n =* 3, *TyrCre^+^* (control); *n =* 3, *RarαDN^LoxP/+^; TyrCre^+^*. Two-tailed unpaired Student’s *t* tests were used for statistics.

We next examined the ENS in mice that express *Cre* in ENCDC from the tyrosinase promoter (*RarαDN^LoxP/+^; TyrCre^+^*) starting at E10.5 (41, 42) when ENCDC normally reach the midgut (3). In these mice, the extent of bowel colonization by ENCDC was similar to controls at E13.5 (Figure 8, A and E). Stomach ENS appeared normal in *RarαDN^LoxP/+^; TyrCre^+^* mice (Figure 8, B and F), but the small bowel and colon were hypoganglionic relative to controls (Figure 8, C, D, G, and H). ENS organization was also abnormal in the small bowel and colon of mutants at E13.5. Instead of the fine network of cells seen in controls, *RarαDN^LoxP/+^; TyrCre^+^* ENS had thick chains of cells (Figure 8, A, C–E, G, and H). At birth, *RarαDN^LoxP/+^; TyrCre^+^* mice appeared healthy but did not feed (absent milk spot, Figure 8I) and consistently died within 12 hours (*n =* 6). To avoid analysis of dying mice, we examined E18.5 ENS. Although the *RarαDN^LoxP/+^; TyrCre^+^; R26R-TdTomato* bowel looked grossly normal (Figure 8J), lineage-marked TdTomato^+^ ENS cells were less dense in the mutant bowel throughout the small intestine and colon (Figure 8K). HuC/D IHC confirmed that enteric neurons were sparse (Figure 8, L, and N–P) and that all neurons in *RarαDN^LoxP/+^; TyrCre^+^; R26R-TdTomato* bowel were TdTomato^+^ (Figure 8, L and M). To determine if blocking RAR impacted neuron subtype ratios, we counted NOS1 and HuC/D double-labeled cells and discovered that more HuC/D^+^ neurons were NOS1^+^ in *RarαDN^LoxP/+^; TyrCre^+^* ENS (Figure 8, Q–S) compared with controls. Collectively, these results show that cell-autonomous RAR signaling is important after E10.5 for ENS patterning, to increase neuron number, and for neuron subtype specification.

### Inactivation of RAR signaling in the RET lineage causes distal bowel hypoganglionosis.

**Figure 9:**
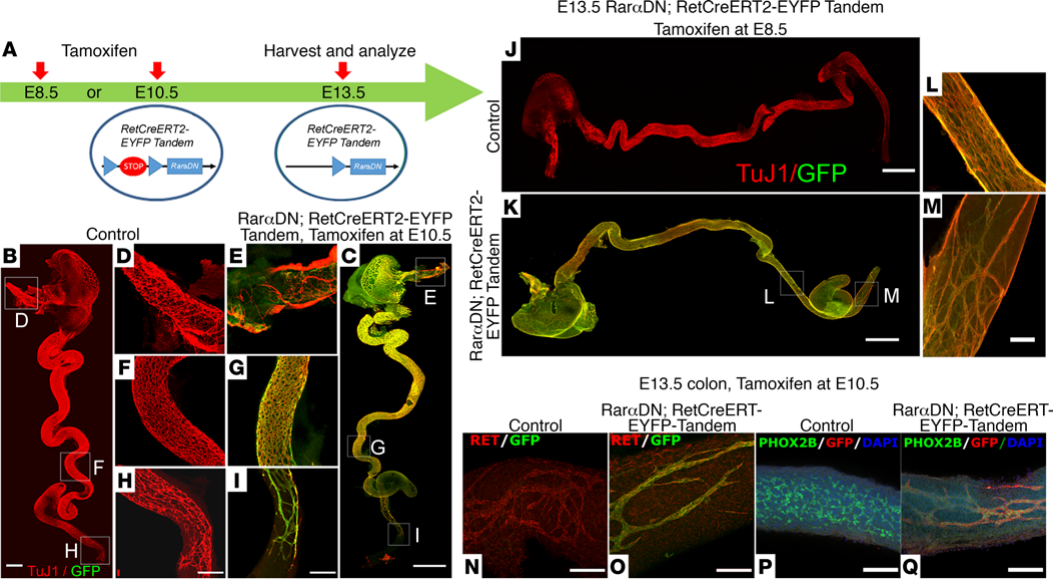
Inactivation of RAR signaling in *Ret* lineage at E8.5 or E10.5. (A) *RarαDN^LoxP/+^; RETCreERT2-EYFP^Tandem^* have *CreERT2* knocked into the *Ret* locus and *ChR2-Lox-Stop-Lox-EYFP* in the nearby *Rosa26* locus on the same chromosome. Tamoxifen activates CreERT2 to induce expression of *RarαDN* and *EYFP*. (**B** and **C**) E13.5 whole bowel from control (*RarαDN^LoxP/+^* that lacks *RETCreERT2-EYFP^Tandem^*) (**B**) *RarαDN^LoxP/+^; RETCreERT2-EYFP^Tandem^* (**C**) after E10.5 tamoxifen treatment. ENS was visualized using TuJ1 (red) and GFP (green) antibodies. (**D**–**I**) Boxes indicate regions of esophagus (**D** and **E**), small bowel (**F** and **G**), and distal colon (**H** and **I**) that are magnified in adjacent images. Scale bars: 1000 μm (**B** and **C**) and 500 μm (**D**–**I**). (**J** and **K**) E13.5 bowel after E8.5 tamoxifen treatment. Scale bar: 1000 μm. (**L** and **M**) Regions of magnified images from *RarαDN^LoxP/+^; RETCreERT2-EYFP^Tandem^* small bowel (**L**) or distal colon (**M**). Scale bar: 100 μm. (**N** and **O**) RET (red) and GFP (green) antibody stained E13.5 colon after E10.5 tamoxifen treatment. (**P** and **Q**) PHOX2B (green), GFP (red), and DAPI (blue) stained E13.5 colon after E10.5 tamoxifen treatment. Scale bars: 100 μm. *n =* 3 in each group.

Sparse ENS in the preceding models might reflect inadequate RET, making it difficult to identify other roles for RAR signaling in ENCDC. Our prior studies suggest that RA is not needed to sustain *Ret* expression in ENCDC that already express *Ret* (26, 27). We therefore decided to use mice expressing *CRE-ERT2* from the *Ret* locus to activate *RarαDN* expression in ENCDC that already express *Ret*. For these experiments, we also needed a CRE-dependent fluorescent reporter (EYFP) to track CRE activity. Because *Ret* is on mouse chromosome 6 near the *ROSA26* locus that drives expression of *RarαDN*, as well as most fluorescent reporters, we bred *RarαDN^LoxP/+^* mice to *RETCreERT2-EYFP^Tandem^* mice to generate *RarαDN^LoxP/+^;* *RETCreERT2-EYFP^Tandem^* and *RarαDN^LoxP/+^* controls. The *RarαDN^LoxP/+^;* *RETCreERT2-EYFP^Tandem^* have undergone recombination so *RETCreERT2* and a CRE-activated EYFP–Channelrhodopsin-2 (ChR2) protein are on the same chromosome. E13.5 control *RarαDN^LoxP/+^* lacking CreERT2 had the bowel fully colonized by ENS precursors after E10.5 tamoxifen treatment (Figure 9, A, B, D, F, and H). E10.5 tamoxifen-treated *RarαDN^LoxP/+^;* *RETCreERT2-EYFP^Tandem^* also had normally patterned ENS in the esophagus and small bowel (Figure 9, A, C, E, and G) but had hypoganglionic ENS in the colon, with thick chains of ENS cells (Figure 9,C and I). We confirmed high levels of CRE-activation in the ENS after tamoxifen treatment and almost no CRE activation in the absence of tamoxifen in these *RETCreERT2-EYFP^Tandem^* mice (Supplemental Figure 3). Because tamoxifen effects may take 12–18 hours, we also tamoxifen treated at E8.5 but found similar phenotypes (Figure 9, J–M). RET and PHOX2B were readily detected in EYFP^+^ ENCDC of E13.5 *RarαDN^LoxP/+^;* *RETCreERT2-EYFP^Tandem^* mice, consistent with our prior studies (26, 27). These data suggest that RAR is needed to activate RET and PHOX2B expression in ENCDC (Figure 4, D–I, and Figure 5, A–F and H) but not to maintain expression once regulatory elements are activated (Figure 9, N–Q). The unusual patterning in the distal colon of tamoxifen-treated E13.5 *RarαDN^LoxP/+^;* *RETCreERT2-EYFP^Tandem^* mice suggests that RAR regulates additional ENS patterning genes in ENCDC.

### Ret lineage loss of RAR signaling profoundly reduced submucosal neuron density and altered cell identity.

**Figure 10:**
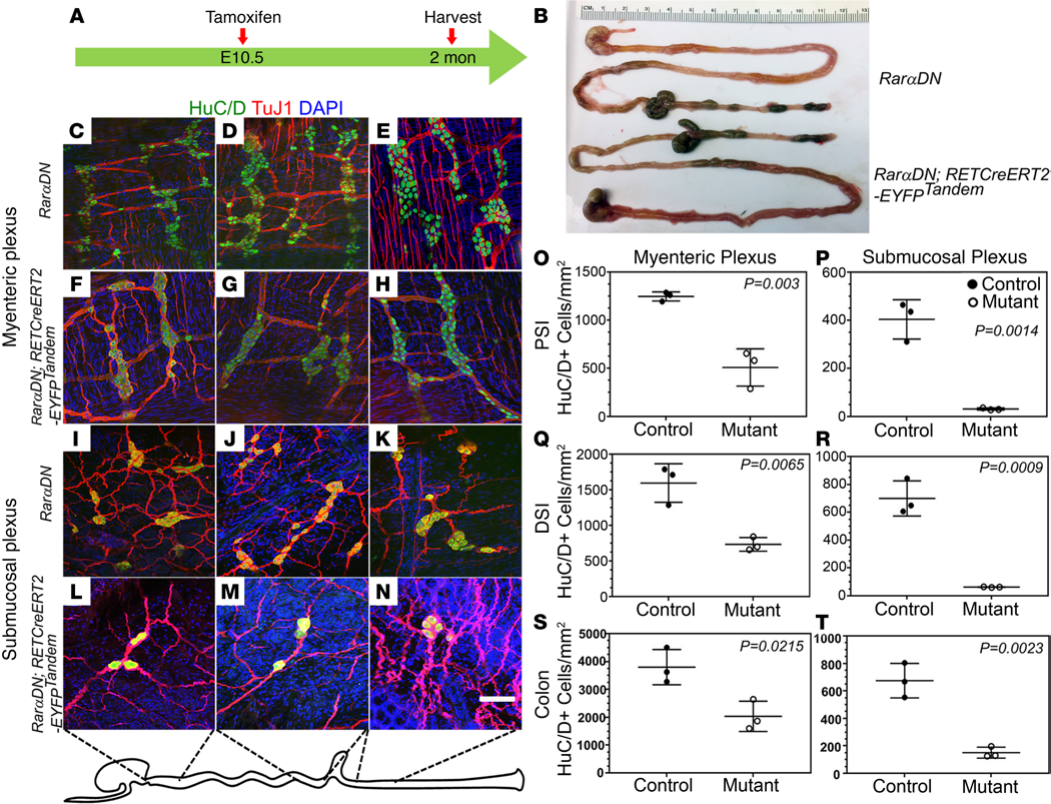
Postnatal ENS phenotype when RAR signaling is inactivated in *Ret* lineage starting from E10.5. (A) Tamoxifen was given to pregnant mice at E10.5 to activate the *RarαDN* transgene, and bowel was harvested at about 2 months of age. (**B**) *RarαDN^LoxP/+^* (control) or *RarαDN^LoxP/+^; RETCreERT2-EYFP^Tandem^* (mutant) bowels appear similar. (**C**–**N**) Myenteric and submucosal plexus in *RarαDN^LoxP/+^* (control) or *RarαDN^LoxP/+^; RETCreERT2-EYFP^Tandem^* (mutant) animals were visualized in whole mounts using HuC/D (green) and TuJ1 (red) antibodies plus DAPI (blue). Scale bar: 100 μm. (**O**–**T**) Quantification of HuC/D^+^ neurons shows about 50% fewer myenteric and about 90% fewer submucosal neurons in *RarαDN-RETCreERt2-EYFP^Tandem^* (mutant) animals compared with *RarαDN^LoxP/+^* (control) (*n =* 3 in each group). Two-tailed unpaired Student’s *t* tests were used for statistics.

*RarαDN^LoxP/+^; RETCreERT2-EYFP^Tandem^* treated with tamoxifen at E10.5 (Figure 10) were born at a normal Mendelian ratio, grew normally, and had a normal-appearing bowel (Figure 10B), permitting analysis of adult ENS. We therefore stained the bowel of 2-month-old mice with HuC/D and TuJ1 antibodies (Figure 10, C–N). Quantitative analysis demonstrated a 50%–65% reduction in myenteric neurons in small bowel and colon and a 90% loss of submucosal neurons in *RarαDN*-expressing mutant mice (Figure 10, O–T). In contrast to neurons, adult enteric glia marked by SOX10 antibody appeared similar in abundance in tamoxifen-treated *RarαDN^LoxP/+^; RETCreERT2-EYFP^Tandem^* and control mice (Supplemental Figure 4A). Interestingly, E10.5 tamoxifen treatment also led to a dramatic increase in NOS1^+^ submucosal neurons in *RarαDN^LoxP/+^; RETCreERT2-EYFP^Tandem^* small bowel (Supplemental Figure 4B), and a mild increase in NOS1^+^ myenteric neurons. These studies confirm distinct age-dependent effects of cell-autonomous RAR signaling in the ENS.

### Transcriptional profiling shows that RARαDN differentially impacts gene expression in E11.5 stomach and E13.5 colon ENCDC.

**Figure 11:**
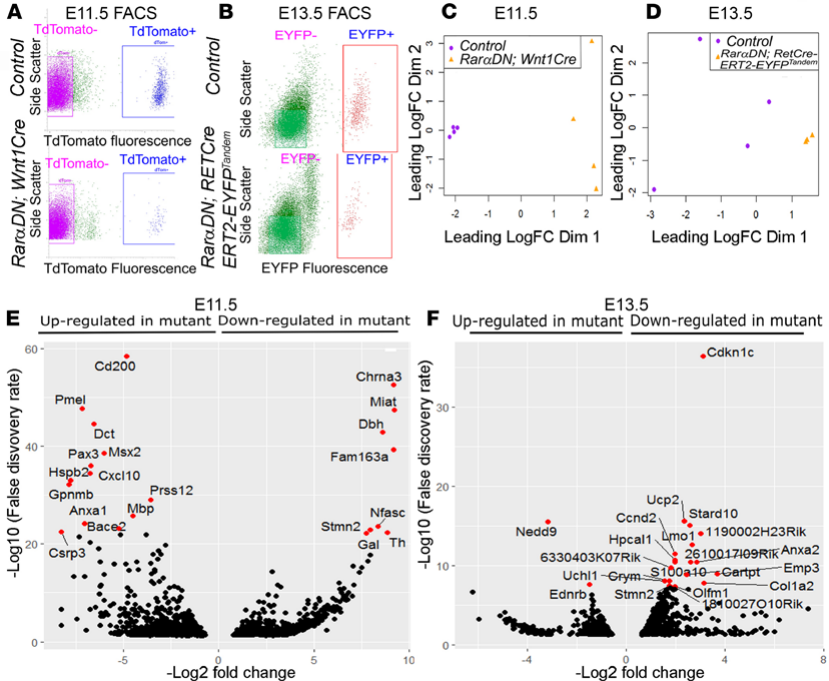
RNA-seq shows that many genes are dysregulated after *RarαDN* expression in the ENS. (**A**) ENCDC were isolated from E11.5 stomach of *RarαDN^LoxP/+^; Wnt1Cre^+^; R26R-TdTomato* and *Wnt1Cre^+^; R26R-TdTomato* (control) mice based on TdTomato fluorescence (*n =* 4 each). (**B**) ENCDC were isolated from E13.5 colon of *RarαDN^LoxP/+^;* *RETCreERT2-EYFP^Tandem^* or *RETCreERT2-EYFP^Tandem^* (control) mice based on EYFP fluorescence after tamoxifen treatment at E10.5 (*n =* 4 each, but 1 *RarαDN^LoxP/+^;* *RETCreERT2-EYFP^Tandem^* failed quality control, leaving *n =* 3 for this genotype). (**C** and **D**) Multidimensional scaling analysis (MDS) of RNA-seq data. (**E** and **F**) Volcano plots show differentially expressed genes.

**Figure 12:**
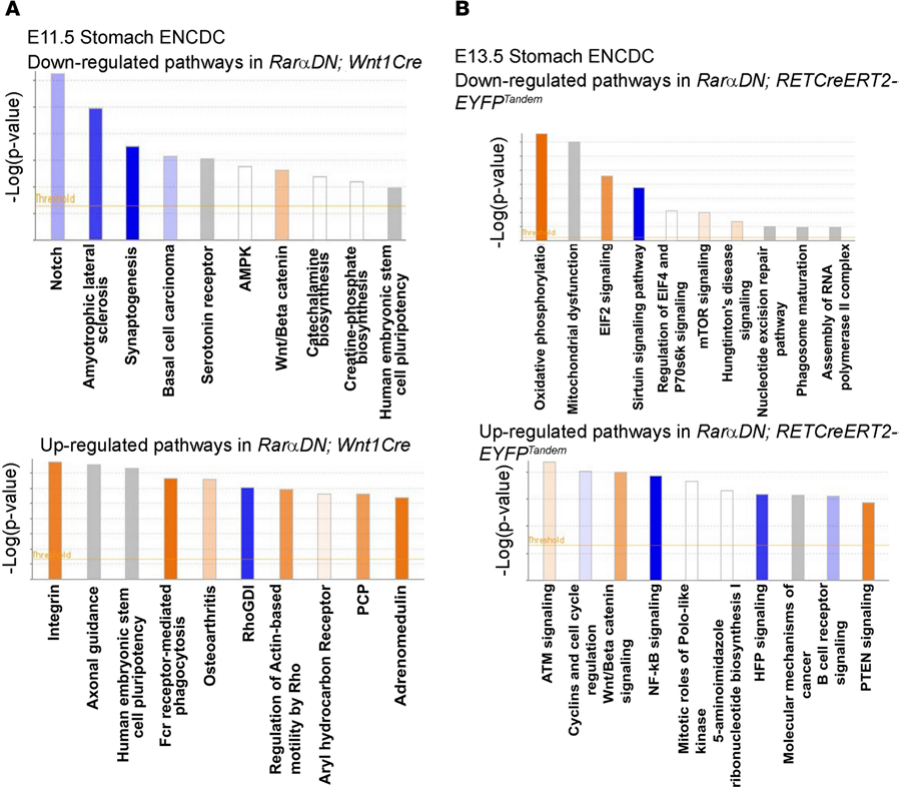
Pathway analysis for genes dysregulated by RARαDN. (**A** and **B**) Ingenuity Pathway Analysis (IPA) of differentially expressed genes (FDR < 0.05) at E11.5 (**A**) and E13.5 (**B**).

Because RAR regulates transcription, we performed pooled-cell RNA sequencing (RNA-seq) to characterize RAR*α*DN-induced changes in ENCDC gene expression. For *RarαDN^LoxP/+^; Wnt1Cre^+^; R26R-TdTomato* mice, we analyzed E11.5 stomach ENCDC because RAR*α*DN eliminated ENCDC beyond the stomach. For *RarαDN^LoxP/+^;* *RETCreERT2-EYFP^Tandem^*, we analyzed E13.5 colon ENCDC after tamoxifen treatment at E10.5 because RAR*α*DN-expressing colon ENCDC had patterning defects. Controls expressed fluorescent protein in ENCDC but lacked *RarαDN*. Flow sorting cleanly separated fluorescent cells from other bowel cell types (Figure 11, A and B). Multidimensional scaling (MDS) showed separate groups of control versus *RarαDN*-expressing ENCDC (Figure 11, C and D). Among approximately 23,000 transcripts at each age, we found 1413 genes differentially expressed between control and *RarαDN^LoxP/+^; Wnt1Cre^+^* ENCDC (FDR < 0.05) at E11.5 (Supplemental Table 1) and 1140 genes differentially expressed between control and *RarαDN^LoxP/+^;* *RETCreERT2-EYFP^Tandem^* (FDR < 0.05) at E 13.5 (Supplemental Table 2), as represented in volcano plots (Figure 11, E and F). The top 20 upregulated and downregulated genes at E11.5 (Supplemental Figure 5A) and E13.5 (Supplemental Figure 5B) and main dysregulated pathways (Figure 12A for E11.5 and Figure 12B for E13.5) suggest that many distinct biological processes are influenced by RAR signaling.

One initially surprising feature at E13.5 was that many hemoglobin genes were less abundant in flow-sorted cells from *RarαDN;* *RETCreERT2-EYFP^Tandem^* compared with controls. We suspect that this occurred because *Ret* is expressed in the hematopoietic stem cell (HSC) lineage, where RAR supports erythropoiesis (43, 44) and hemoglobin genes are normally expressed at high levels. Consistent with this hypothesis, some *RETCreERT2-EYFP^Tandem^*–lineage cells in the colon were stained with TER119 (erythroid lineage) and CD31 (endothelial, platelet, and leucocyte lineage) antibodies (Supplemental Figure 6). To define a gene set clearly linked to the ENS, we compared differentially expressed genes in *RarαDN;* *RETCreERT2-EYFP^Tandem^* and *RarαDN; Wnt1Cre; TdTomato* mice. In total, 594 genes were differentially regulated versus age-matched control cells in both mouse lines (Supplemental Figure 7 and Supplemental Table 3). Of these, a core set of 115 genes were regulated in the same direction by RAR*α*DN in ENCDC at E11.5 and E13.5 (Supplemental Tables 4 and 5). Gene enrichment pathway analysis of the aforementioned 2 sets of genes showed many pathways related to neuron development (Supplemental Figure 7). To validate RNA-seq data, we selected 2 differentially expressed genes of ENCDC at E11.5 (*Stmn2* and *Pax3*) and performed quantitative PCR (qPCR), which confirmed differences predicted by RNA-seq (Supplemental Figure 8). The RNA-seq data for *Ret* and *Phox2B* in E11.5 stomach also correlate well with our IHC for RET and PHOX2B protein (Figure 4, Figure 5, and Supplemental Figure 9).

## Discussion

RA regulates activity of the RAR/RXR transcription factor family to alter gene expression and influence many aspects of development (33). Prior studies using constitutive KO mice, RAR antagonist, stem cells, or vitamin A depletion in mice and rats show critical roles for RA in the developing ENS (21–32). For example, loss of the RA biosynthetic enzyme RALDH2 causes extensive bowel aganglionosis in *Raldh2^–/–^* mice (29), while *Raldh1^–/–^* and *Raldh3^–/–^* mice have milder ENS defects (25). The *Raldh2^–/–^* phenotype probably occurs because RA is needed in vagal paraxial mesoderm starting at E9 in mice (or E1.5 in avians) to induce *Ret* expression in ENCDC as these cells migrate from neural tube to bowel (24). In contrast, when RAR signaling was blocked by the chemical pan-RAR antagonist BMS493 at E11.5 in organ culture (26) or at E12.5 in dissociated cell culture (27), RET expression in ENCDC appeared unaffected by RAR inhibition. Nonetheless, RAR inhibition at these later stages impaired ENCDC proliferation, bowel colonization, neurite growth, neuronal differentiation, and ENS patterning (23, 26, 27). Consistent with these observations, nutritional deficiency in the RA precursor vitamin A causes distal bowel aganglionosis in mice, mimicking human Hirschsprung disease (HSCR) (26), and gene variants in people with HSCR may alter RA signaling (45, 46). These studies suggest that RA signaling could impact ENS development in many ways, depending on the timing and severity of the RA signaling defect.

Prior strategies did not define the cell types influenced by RA signaling. To determine how cell-autonomous RAR signaling affects the ENS lineage, we used a potent CRE-inducible dominant-negative RAR transgene (RARaT403) that blocks all 3 RAR receptors (i.e., *RarαDN^LoxP/+^* mice) (47). By breeding to several different CRE-expressing mouse lines, we induced RARaT403 (RAR*α*DN) expression selectively within ENCDC at specific times during development. Our *RarαDN^LoxP/+^; Wnt1Cre^+^* studies show that cell-autonomous RAR signaling within ENCDC is required to activate *Ret* expression early in development, consistent with data from zebrafish (21) and avian models (24). RAR signaling is also needed to activate *Ret* in the ureteric bud, suggesting that similar cell-autonomous mechanisms may control *Ret* expression in the kidney and developing ENS (34, 48). In contrast, when CRE expression is driven by *Ret* regulatory elements, *RarαDN* induction did not lead to loss of RET protein in ENCDC, suggesting that RA is needed to turn on *Ret* but not to maintain *Ret* expression, consistent with our prior BMS493 data (26, 27). We were surprised to discover that PHOX2B protein was also undetectable in ENCDC of *RarαDN^LoxP/+^; Wnt1Cre^+^* mice. Loss of PHOX2B could, by itself, explain the loss of RET in *RarαDN^LoxP/+^; Wnt1Cre^+^* ENCDC (40), but we are unable to find evidence that PHOX2B regulates RET in the kidney, suggesting that *Ret* is regulated by RA via additional PHOX2B-independent mechanisms, as supported by prior studies (30, 45, 49).

There were many other interesting observations. First, the thick chains of enteric neurons seen in the distal bowel of *RarαDN^LoxP/+^;* TyrCre^+^ and *RarαDN^LoxP/+^; RETCreERT2-EYFP^Tandem^* mice closely resemble the colon ENS patterning defect we observed in BMS493-treated fetal gut organ cultures (26). This suggests that the normal dispersion of enteric neurons into small colon ganglia is RA dependent. Second, in fetal *RarαDN^LoxP/+^; SOX10Cre^+^* mice, we saw some clustered small bowel enteric neurons near extrinsic nerve fibers but far from the more proximal ENS cells. These clusters closely resemble Schwann cell–derived ENS described by Uesaka et al., but the ENCDC we observed were restricted to small regions of fetal bowel (10). Alternatively, these cells might originate from the “mesenteric neural crest cells” recently described by Yu et al. that they hypothesize contribute to human skip segment HSCR (8). Third, vagus nerves at E11.5 in *RarαDN^LoxP/+^; Wnt1Cre^+^* mice occupied a smaller area of the stomach compared with control animals. Since vagus nerve fibers were not TdTomato labeled in *Wnt1Cre; R26R-TdTomato* mice, this suggests reciprocal interactions between migrating *Wnt1Cre* lineage ENCDC and growing vagal fibers. Fourth, in addition to approximately 80% reduction in total neuron number in the ENS of *RarαDN^LoxP/+^; TyrCre^+^* mice, there was a striking increase in the percentage of enteric neurons that express NOS1 (nitric oxide synthase). We confirmed that these NOS1^+^ neurons had expressed CRE using an *R26R-TdTomato* reporter. This suggests that NOS1^+^ neuron differentiation is less dependent on RAR signaling than other neuron subtypes or that RAR turns off *Nos1* expression in some enteric neuron subtypes. Finally, we found an almost complete loss of submucosal neurons in *RarαDN^LoxP/+^; RETCreERT2-EYFP^Tandem^* mice treated with tamoxifen at E10.5, suggesting a critical role for RA signaling in radial migration of ENCDC to form submucosal plexus. The normal postnatal growth of these tamoxifen-treated *RarαDN^LoxP/+^; RETCreERT2-EYFP^Tandem^* mice and normal appearance of the adult bowel suggests that a loss of approximately 90% of submucosal neurons is well tolerated, at least in mice. This is interesting, in part, because we know little about mechanisms controlling radial migration of ENS precursors to the submucosal plexus, with prior studies implicating only GDNF and netrin/DCC signaling in this process (50, 51). These observations highlight the remarkable range of ENS abnormalities that may occur when RAR signaling is inadequate and are consistent with the observation that low serum vitamin A is associated with increased constipation in children with autism (52).

One concern is that we did not evaluate ENS biology in every possible control group, so we cannot exclude some effects of tamoxifen, *Cre* alleles, fluorescent reporters, or the *RarαDN^LoxP/+^* allele in isolation. For example, a recent study clearly shows that *Wnt1Cre^+^; R26R-TdTomato^+^* increases the severity of the *Ednrb^–/–^* ENS phenotype (8). We also were underpowered to examine the effect of sex on ENS phenotypes, an issue that could be important for some of the milder phenotypes we examined.

One advantage of our strategy is that CRE-dependent expression of RAR*α*DN and a fluorescent reporter in the same cells facilitated flow sorting and RNA-seq to identify RAR targets. Our analyses identified thousands of differentially expressed genes in RAR*α*DN-expressing versus control ENCDC at E11.5 and E13.5 with 115 genes regulated in the same way by RAR*α*DN at each age. Interpreting these data is complicated, since RAR*α*DN induces changes in cell type ratios, in addition to changes in gene expression in individual cells. Furthermore, it is not clear if the induced changes occur because of direct effects on RAREs or if they reflect more global effects on the differentiation state of the sequenced cells. For example, loss of PHOX2B in E11.5 ENCDC should change the expression of many genes, independent of effects on RAR activity.

As one strategy to further define the role of RAR signaling, we narrowed our lists to include only differentially expressed genes (FDR < 0.2) where expression changed in the same direction at E11.5 and E13.5 for RAR*α*DN^+^ cells (Supplemental Tables 4 and 5). At E11.5, there were 55 genes expressed at higher levels in WT ENCDC than in RAR*α*DN^+^ ENCDC. Thirty-five of these genes had easily identified functions in neurons. Thirteen genes at E11.5 were more abundant in RAR*α*DN^+^ ENCDC than in WT ENCDC. This list included *Rest*, an epigenetic master negative regulator of neurogenesis (53, 54), and *Bmp4*, a gene with complex roles in ENS patterning (55–62). At E13.5, our list included 44 genes more abundant in WT than in RAR*α*DN^+^ cells. Twenty-nine of these genes had easily identified roles in neurons. Genes more abundant in RAR*α*DN^+^ than in WT cells at E13.5 included *Rest*, *Sox10*, and *Ets1*, consistent with a role for RAR in ENS neurogenesis. The SOX10 transcription factor is essential for ENS development (63), at least in part because SOX10 activates RET expression (64, 65). However, as multipotent ENCDC differentiate into enteric neurons, SOX10 expression is lost, while enteric glia continue to express SOX10 through adulthood (3). ETS1 enhances *Sox10* expression and is essential to make radial glia in *Xenopus* (66). Consistent with these mRNA findings, SOX10^+^ cells were abundant in myenteric and submucosal plexus of adult *RarαDN*-expressing mice, even though *RarαDN^LoxP/+^; RETCreERT2-EYFP^Tandem^* had an approximately 90% reduction in submucosal neurons and the enteric glia are derived from RET^+^ ENCDC (67). In addition, many Wnt ligands (Wnt1, Wnt2, Wnt4, and Wnt10a) were highly differentially expressed in E11.5 ENCDC of *RarαDN^LoxP/+^; Wnt1Cre^+^* versus control. Since Wnt signaling plays important roles in many aspects of neural development (68–70), RAR might alter Wnt signaling to regulate ENCDC development. Collectively, these data suggest that cell-autonomous RAR signaling directs neurogenesis in the ENS from multipotent ENCDC and that cell-autonomous RAR signaling has distinct effects at many stages of ENS development. Our observations further suggest that maternal retinoid status during pregnancy and postnatal vitamin A deficiency or excess could have long-term effects on ENS structure and function. This may be important because vitamin A deficiency is a common problem in many regions of the world (71).

## Methods

### Mouse strains.

*RarαDN^Loxp–STOP–LoxP/+^* (previously described in ref. 34) were rederived from sperm into C57BL/6J background by the CHOP transgenic core. *H2az2(Tg[Wnt1cre]11Rth)* (Research Resource Identifiers [RRID]: 2386570_JAX:003829; referred to as *Wnt1Cre*), *TyrCre* (RRID: MGI:358524_JAX:029788), *SOX10Cre* (MGI:3586900_JAX:025807), *Rosa26^EYFP^* (*Gt[ROSA]26Sortm1[EYFP]Cos,* MGI: 2449038, RRID:IMSR_EM:09668), and *Gt(ROSA)26Sortm9(CAGtdTomato)Hze* (RRID:IMSR_JAX:007909; referred to as *R26R-TdTomato*) were from The Jackson Laboratory and maintained in C57BL/6J background. *RETCreERT2-EYFP^Tandem^* mice, a gift from Wenqin Luo (University of Pennsylvania), were generated by breeding *RETCreERT2* (RRID: MGI:4437245) to mice with *ChR2-Lox-Stop-Lox-EYFP* in the *Rosa26* locus (72). Mouse *Ret* and *Rosa26* loci are close together (about 5 million bp), but *RETCreERT2-EYFP^Tandem^* recombined so that they are on the same chromosome. *Ret^TGM/TGM^* (*Ret^–/–^* allele *Ret^tm1Jmi^*) on C57BL/6J were previously described (73). A complete list of mouse strains appears in Supplemental Table 6, Genotyping was performed using previously published and potentially novel primers (Supplemental Table 7).

### Mouse husbandry.

Mice had a 12-hour light-dark cycle with free access to food and water. Additional details based on ARRIVE guidelines (74) are in Supplemental Table 8. For timed pregnant mating, *RarαDN* female mice were bred with *Cre*-expressing male mice in the late afternoon. Successful mating was confirmed by vaginal plug the next morning, which was designated E0.5.

### EdU incorporation assay.

EdU analyses were performed using a Click-iT EdU imaging kit (Thermo Fisher Scientific, C10337). Timed pregnant animals (E12.5) were injected (i.p.) with EdU (12.5 μg/gm body weight). Tissues were dissected 4 hours after EdU and fixed (4% paraformaldehyde [PFA], 30 minutes at room temperature) before staining, using manufacturer’s protocol.

### Whole mouse embryo imaging using 3DISCO clearing.

3DISCO imaging was performed as described (38) with modification. Briefly, E10.5 embryos from timed-pregnant females were washed in PBS (pH 7.4), fixed (4% PFA, 1 hour, room temperature), and then incubated in SOX10 (1:200, Novus Biologicals, AF2864) and TuJ1 (1:2000 dilution, Covance, PRB-435P, RRID:AB_2313773) antibodies in 1× PBS with 0.5% triton X-100 (PBST) and 5% normal donkey serum (NDS) (4°C, constant rocking, 72 hours). After washing 4 times (PBS, room temperature, 15 minutes per wash), embryos were incubated in secondary antibodies and Hoechst 33342 for nuclear staining (2 μg/mL, Invitrogen, catalog H3570) in PBST with 5% NDS (4°C, overnight) and then washed 4 times (PBS, room temperature, 15 minutes per wash) and subjected to 3DISCO clearing using a series of tetrahydrofuran (MilliporeSigma, 401757) incubations (room temperature, 50%, 70%, and then 80% in double-distilled water, 60 minutes each, with rocking), followed by incubation in 100% tetrahydrofuran (3 × 20 minutes, room temperature, rocking). Embryos were then incubated overnight in dibenzyl either (MilliporeSigma, 108014-1KG) at room temperature and mounted in dibenzyl ether before confocal imaging.

### IHC.

For whole-mount staining, fetal bowel was fixed as a tube. Adult bowels were opening along mesentery and pinned flat to Sylgard before fixation. Bowels were fixed with 4% PFA (40 minutes, room temperature, rocking), washed (4 × 10 minutes, PBS, room temperature), blocked (5% NDS in PBST, room temperature, 1 hour), and then incubated overnight (4°C) with primary antibodies in 5% NDS in PBST. Secondary antibody staining, unless otherwise specified, was done by incubating samples in a 1:400 dilution of the appropriate antibody (1 hour, 25°C). All the primary and secondary antibodies are listed in Supplemental Table 9.

### RNA-seq analyses.

E11.5 stomach ENCDC were isolated from *RarαDN^LoxP/+^; Wnt1Cre^+^; R26R-TdTomato* and *Wnt1Cre^+^; R26R-TdTomato* (control) mice. E13.5 colon ENCDC were isolated from *RarαDN^LoxP/+^;* *RETCreERT2-EYFP^Tandem^* or *RETCreERT2-EYFP^Tandem^* (control) mice after treatment with tamoxifen (oral gavage, 233 mg/kg body weight dissolved in corn oil) at E10.5. Individual E11.5 stomach and E13.5 colon were incubated with collagenase (MilliporeSigma, C0130, 0.2 mg/mL) and dispase (MilliporeSigma, 494207800, 0.2 mg/mL) in Ham’s F12 media (Thermo Fisher Scientific, 11765054) with 1% BSA (37^o^C, 5% CO_2_ incubator, 20 minutes) and then triturated by pipetting up and down 15 times using a P1000 pipette tip before passing through a 30 μm strainer (Corning, 352340). Cells with TdTomato or EYFP fluorescence were purified by flow sorting (MoFlo Astrios high speed cell sorter, 100 μm nozzle), pelleted by centrifugation (500*g*, 4°C, 5 minutes), washed once with PBS, and frozen immediately at –80°C until analysis. RNA isolation, library preparation, and RNA-seq analysis were performed by University of Pennsylvania Next-Generation Sequencing Core. RNA extracted using the GenElute Single Cell RNA Purification Kit (MilliporeSigma, RNB300) was analyzed on a 2100 Agilent Bioanalyzer using an RNA 6000 Pico Kit (Agilent, 5067-1513). The libraries were made using the combination of the Clontech (now Takara), SMART (cDNA synthesis and amplification), and Illumina Nextera XT (cDNA to library) kits. Libraries were then single-end sequenced on an Illumina HiSeq 4000, using Sequencer Software Real-Time Analysis (RTA) 2.7.7 and Illumina HiSeq Control Software (HCS) HD 3.4.0.38. Reads were aligned to remove repeat sequences and ribosomal RNA reads and then processed using RNA-seq unified mapper (RUM) package (75). RUM files were visualized in the TessLA browser for down-stream analyses. MDS analysis, volcano plot, and heatmaps were made on R 4.0.2 platform using limma, edgeR, Glimma, gplots, org.Mm.eg.db, and RColorBrewer. We used ggrepel, ggplot2, and reshape to generate Volcano plots, and we used tidyr, gplots, and ggplot2 to generate heatmaps. Gene expression pathway analyses were performed using Ingenuity Pathway Analysis (IPA) package (Qiagen) and Metascape Gene Annotation & Analysis Resource (76). A Venn diagram was generated using the Draw Venn Diagram software of Bioinformatics & Evolutionary Genomics, VIB/UGent, Belgium. The RNA-seq raw data have been deposited in the GEO NIH data repository under the accession no. GSE165344.

### Real time PCR.

E11.5 stomach ENCDC of *RarαDN^LoxP/+^; Wnt1Cre^+^; R26R-TdTomato* and *Wnt1Cre^+^; R26R-TdTomato* (control) mice were isolated via the same protocol as for RNA-seq analysis. RNA was purified using PicoPure RNA isolation kit (Arcturus, KIT0202). cDNA was made by using First-strand cDNA synthesis using superscript II RT kit (Invitrogen, 18064-022) with Oligo(dT)12-18 primer (Invitrogen, 18418012). Quantitative PCR (qPCR) was performed using SYBR Green qPCR mixture (MilliporeSigma, S4438). All primers have been previously described and listed in Supplemental Table 10. Ct values were normalized to *Gapdh* mRNA.

### Confocal microscopy.

High-resolution 3-dimensional images were acquired with a Zeiss LSM 710 confocal microscope using Zeiss ZEN 2.3 SP1 FP3 (black) (Version 14.0.18.201; Zeiss) software and a 20×/0.8 air or 63×/1.4 oil DIC M27 Plan-Apochromat objective, except for whole bowel E12.5 and E13.5 imaging, which employed a 5× objective and the ZEN Tile Scan function. Image stitching employed ZEN software. *Z*-stacks were created with ImageJ/FIJI. Increments between each slice were 1 μm, except for scanning the whole E10.5 embryo, where increments between slices were 3 μm. Videos of *Z*-stacks were created with the animation function of Imaris 9.02.

### Image analysis.

Early embryonic, perinatal, and adult mouse bowel cell counting used > 5 randomly selected ×20 fields per animal in each region and FIJI CellCounter module (NIH). Investigators were blinded to genotype when comparing *RarαDN* mutant and control mice.

### Statistics.

All experiments include at least 3 replicates. Statistical analyses employed GraphPad Prism 7 software. Data normality analyses employed the Shapiro-Wilk test. Comparisons between groups used 2-tailed unpaired Student’s *t* tests. *P* < 0.05 is considered statistically significant. Data show mean ± SEM.

### Study approval.

Mouse studies were performed with approval from the Institutional Use and Care Committee at The Children’s Hospital of Philadelphia (CHOP) Research Institute (IAC 19-001041) and the Animal Studies Committee of Washington University School of Medicine in St. Louis (no. 20120130).

## Author contributions

TG and ROH led this study and wrote the manuscript. TG, ECWJ, RS, JBA, and ROH designed research studies, conducted experiments, acquired data, analyzed data, and edited the manuscript.

## Supplementary Material

Supplemental data

Supplemental Table 1

Supplemental Table 2

Supplemental Table 3

Supplemental Table 4

Supplemental Table 5

Supplemental Table 6

Supplemental Table 7

Supplemental Table 8

Supplemental Table 9

Supplemental Table 10

Supplemental Video 1

Supplemental Video 2

## Figures and Tables

**Table 1:**
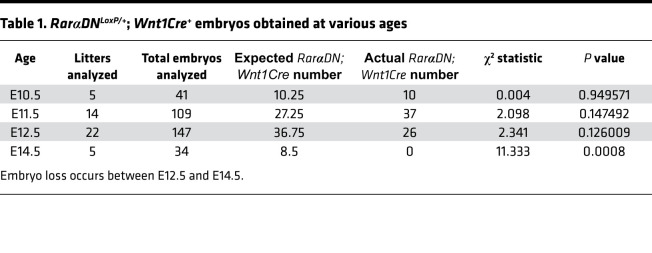
*RarαDN^LoxP/+^*; *Wnt1Cre^+^* embryos obtained at various ages

## Version 1 04/13/2021

In-Press Preview

## Version 2 05/24/2021

Electronic publication
