# Supplementary material for: Cell-autonomous retinoic acid receptor signaling has stage-specific effects on mouse enteric nervous system
Source: JCI Insight. 2021 May 24;6(10):e145854. doi: 10.1172/jci.insight.145854 (PMC8262371; doi:10.1172/jci.insight.145854)
Supplement: Supplemental data [file jciinsight-6-145854-s157.pdf]

Supplemental Figure 1

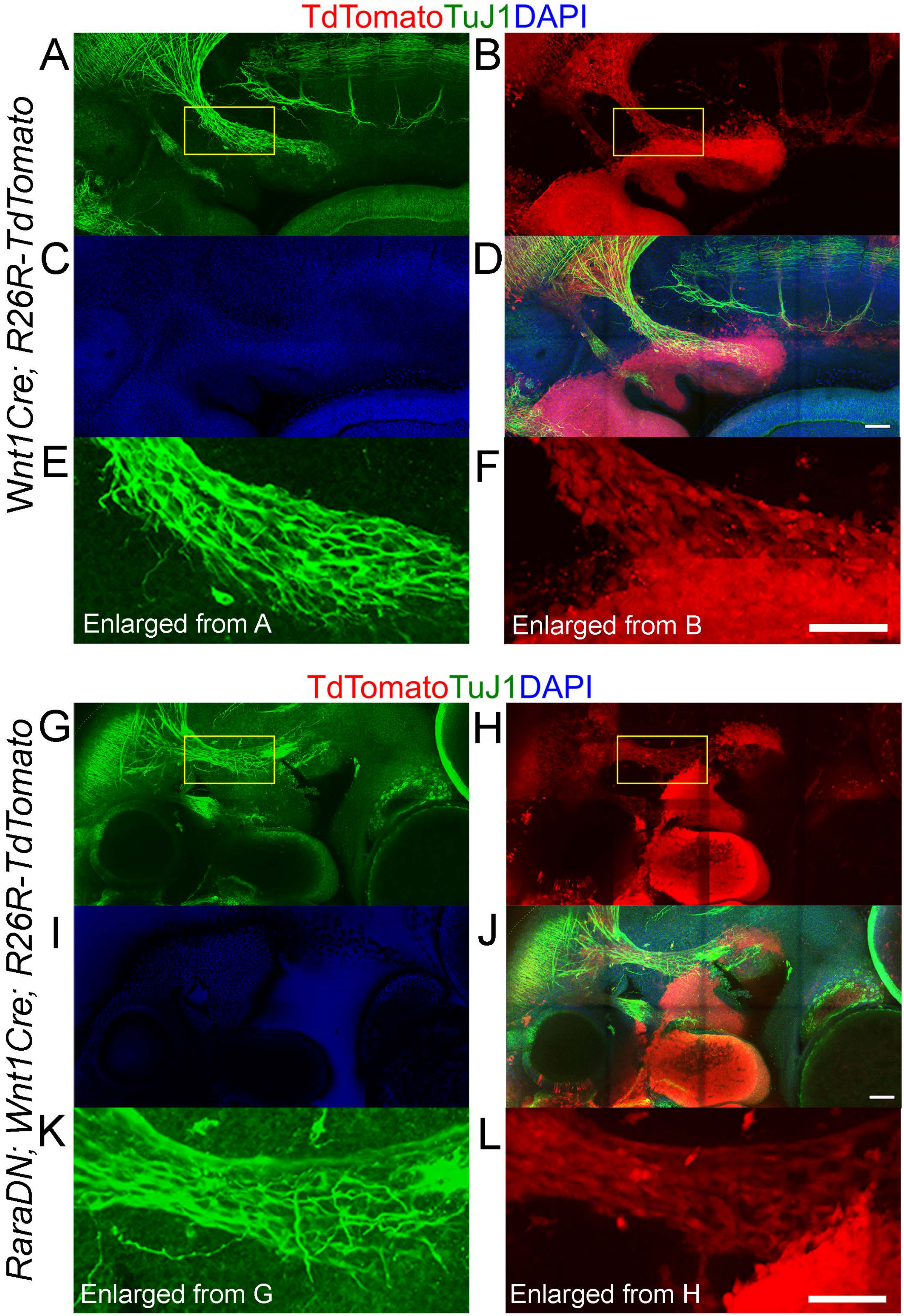

Supplemental Figure 2

E12.5 *SOX10Cre; R26R-TdTomato*

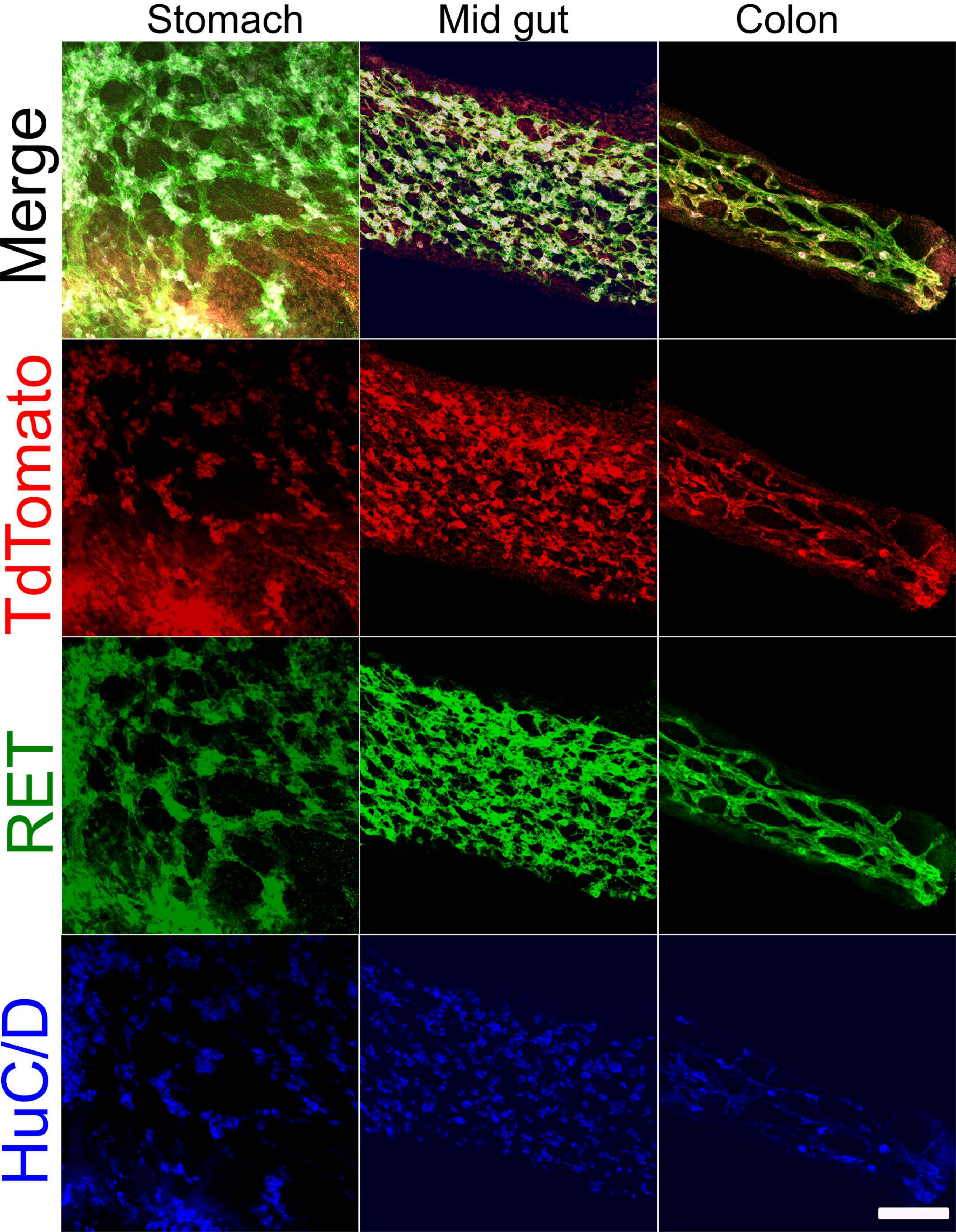

Supplemental Figure 3

RETCreERT2-EYFP^{Tandem}

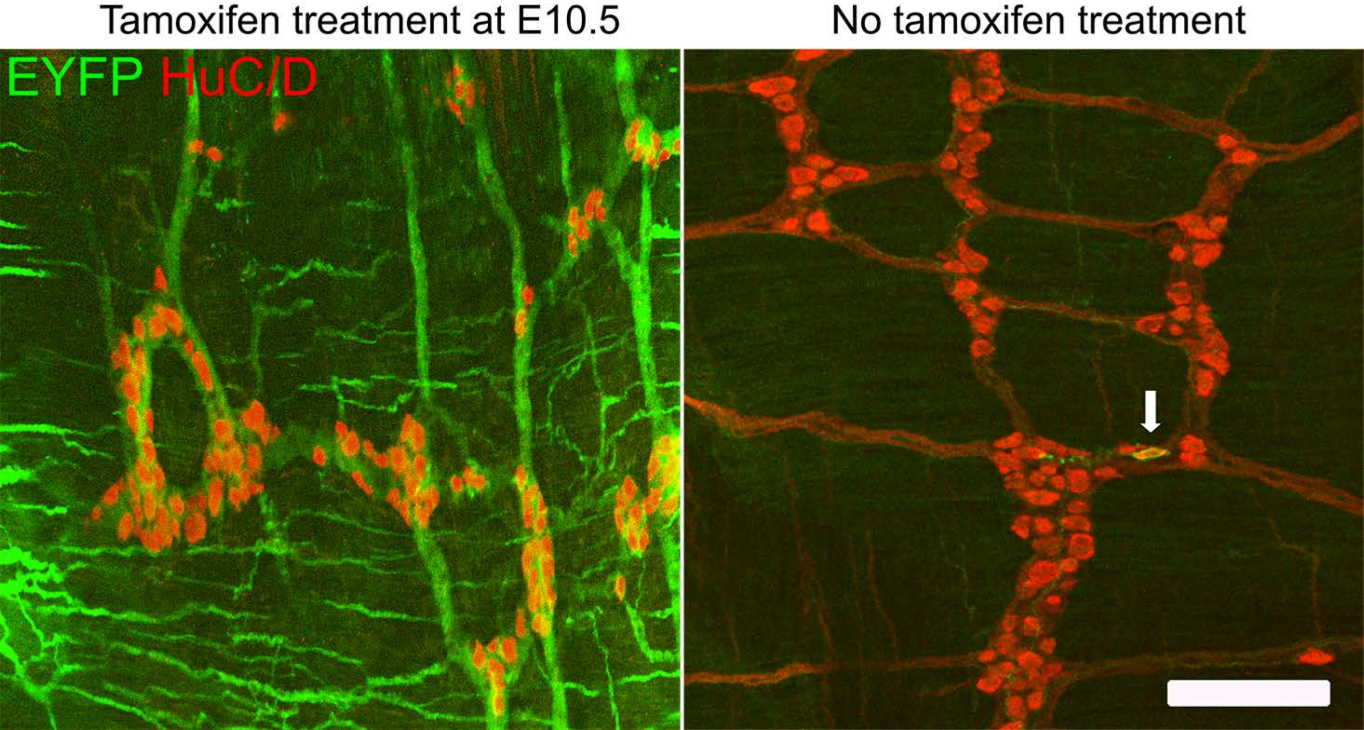

Supplemental Figure 4

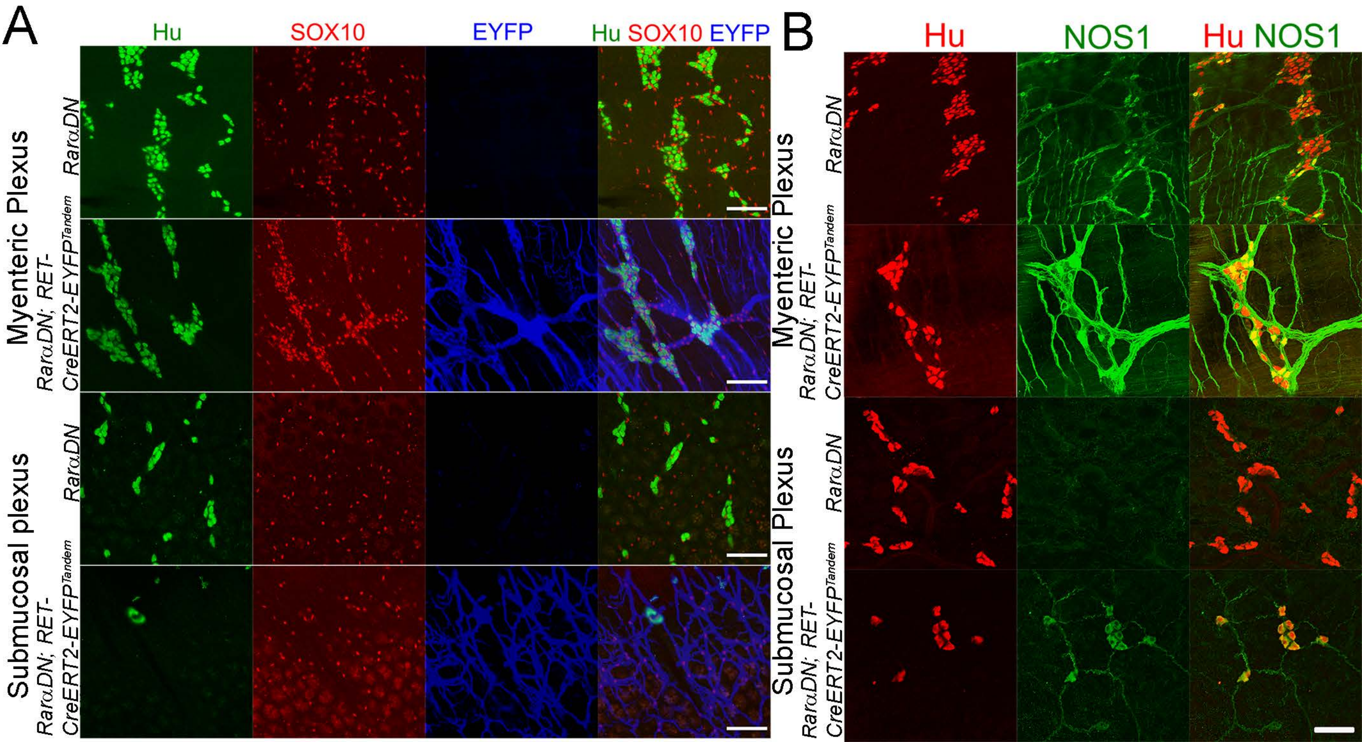

Supplemental Figure 5

A E11.5 Stomach ENCDC

Top 20 genes less abundant in mutant

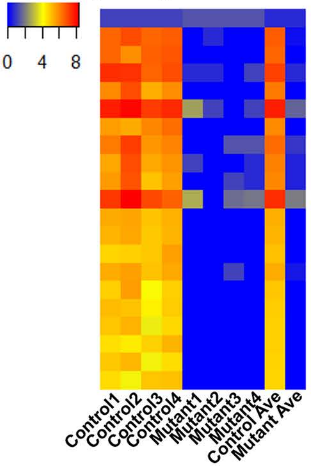

Gene	Fold change (log)	FDR
Miat	-9.2	3.7E-48
Fam163a	-9.2	4.9E-40
Chrna3	-9.2	2.9E-53
Th	-8.8	5.3E-23
Dbh	-8.6	1.5E-43
Nfasc	-8.4	2.5E-24
Stmn2	-8.0	1.1E-23
Rgs4	-7.9	2.oE-18
Stmn3	-7.8	3.3E-17
Gal	-7.8	7.4E-23
Chrna5	-7.5	5.4E-16
Btbd17	-7.4	7.0E-15
Adcyap1r	-7.1	2.2E-12
Mab21l2	-7.1	3.8E-16
Nap1l2	-7.0	4.2E-10
Nrsn1	-6.8	7.5E-11
Slc18a3	-6.8	2.1E-09
Myt1	-6.7	2.8E-10
St18	-6.7	2.9E-09
Frmpd1	-6.6	2.3E-09

Top 20 genes more abundant in mutant

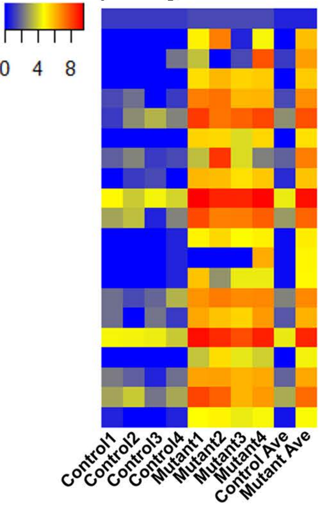

Gene	Fold change (log)	FDR
Bhlhe40	8.3	2.1E-07
Afp	8.3	0.00037
Csrp3	8.3	3.0E-23
Hspb2	7.9	1.7E-35
Gpnmb	7.9	6.9E-33
Slc45a2	7.8	4.2E-14
Hspa1a	7.6	2.1E-05
Trim63	7.5	3.4E-22
Pmel	7.3	1.8E-48
Anxa1	7.2	6.1E-25
Cxcl14	7.1	4.7E-14
Serpina6	7.0	0.0049
Trpm1	7.0	1.6E-07
Pax3	6.8	1.5E-39
Tyr	6.8	5.4E-21
Dct	6.8	3.3E-35
Wnt1	6.7	2.4E-45
Cxcl10	6.7	7.2E-08
Cryab	6.5	8.9E-20
C2	6.5	1.6E-10

B E13.5 Colon ENCDC

Top 20 genes less abundant in mutant

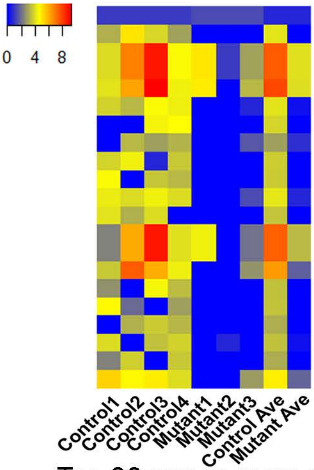

Gene	Fold change (log)	FDR
Hba.x	-7.4	2.8E-05
Hba.a1	-6.2	0.0006
Hba.a2	-6.0	0.021
Beta.s	-6.0	0.021
Npy	-5.7	0.0211
Lgals3	-5.6	0.00019
Scara5	-5.6	0.03
Chodl	-5.5	7E-05
Ptger3	-5.4	0.0074
Wnt11	-5.2	0.013
Hs3st2	-5.2	0.00011
Hbb.b2	-5.2	0.015
Hbb.b1	-5.1	0.02
Hbb.y	-5.1	0.02
Gabra5	-5.1	0.02
Klhl4	-4.9	0.038
Cd300a	-4.7	0.00021
Gpr75	-4.7	0.031
Ano4	-4.6	
Scg2	-4.6	

Top 20 genes more abundant in mutant

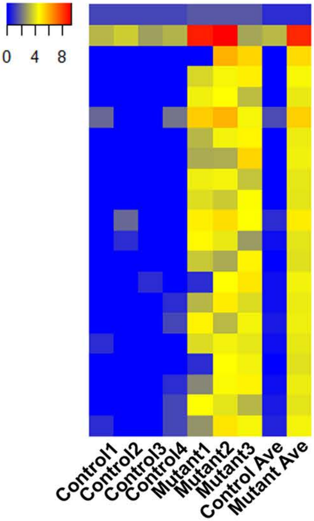

Gene	Fold change (log)	FDR
Slc7a11	6.2	2.1E-07
Slc40a1	6.0	0.00057
Galnt3	5.1	0.0002
Fzd4	4.9	0.0006
Sema3c	4.9	6.7E-05
X9130019O22Rik	4.9	0.00052
Bhlha15	4.9	0.0016
Bmp4	4.9	0.0007
Lifr	4.8	0.0007
X1700019G17Rik	4.7	0.00047
Adam22	4.6	0.0041
Igsf9b	4.6	0.0021
Enpp2	4.6	0.009
Mbnl3	4.6	0.0013
Prkcq	4.6	0.0016
Oprl1	4.6	0.00083
Trpc6	4.5	0.0071
Cdh24	4.5	0.0023
Parp9	4.5	0.003
Rapgef3	4.4	0.003

Supplemental Figure 6

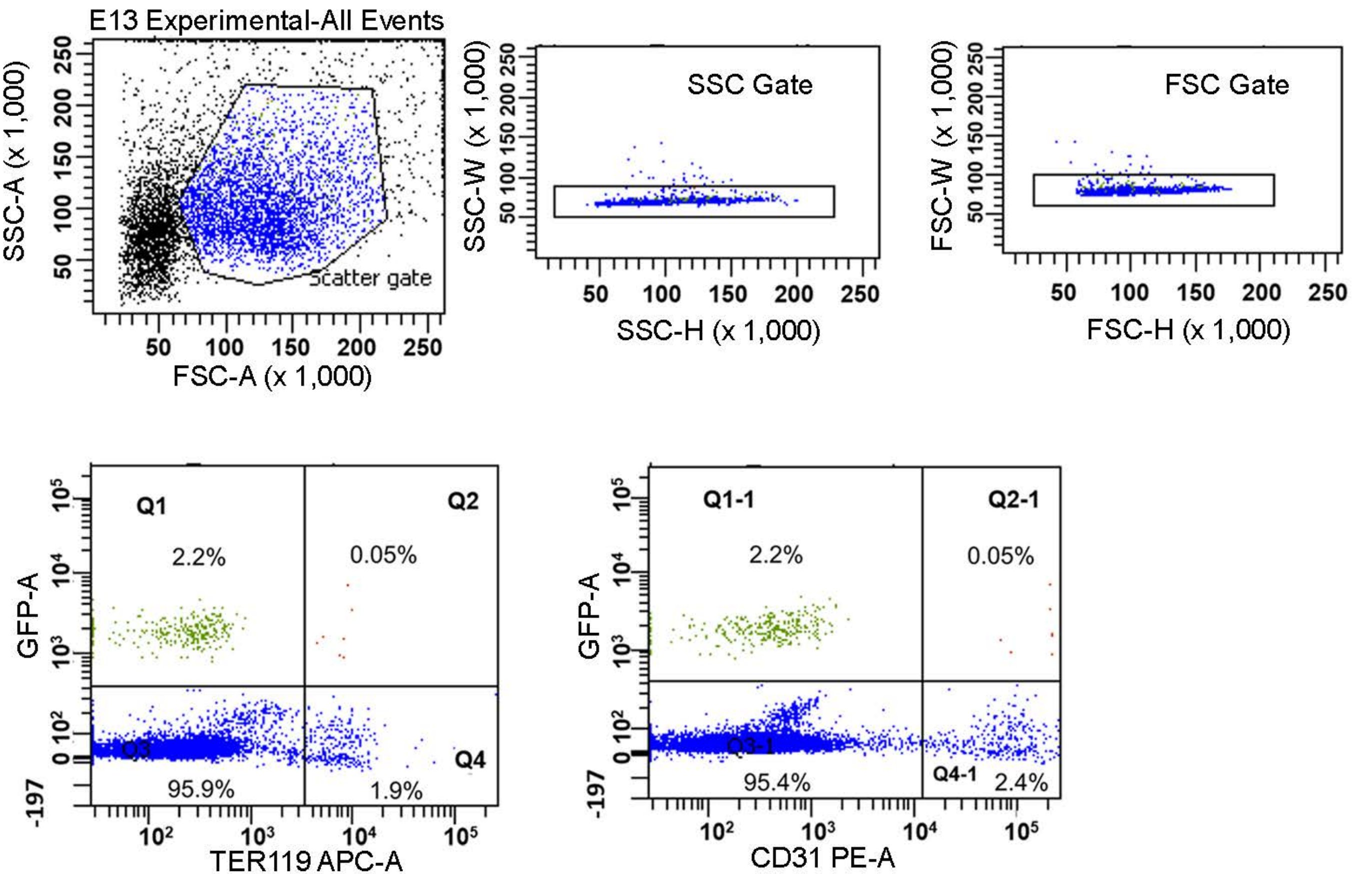

Supplemental Figure 7

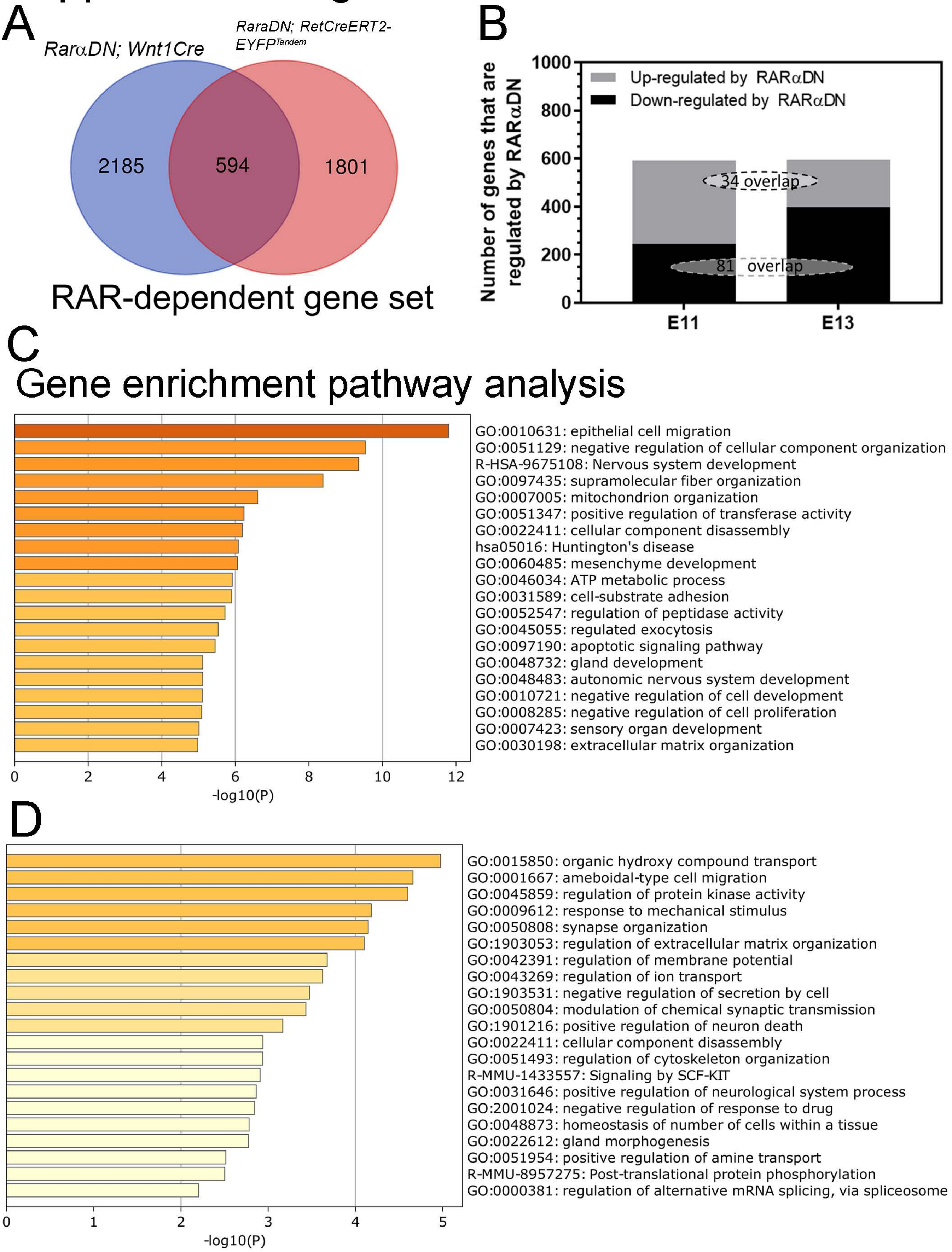

Supplemental Figure 8

A

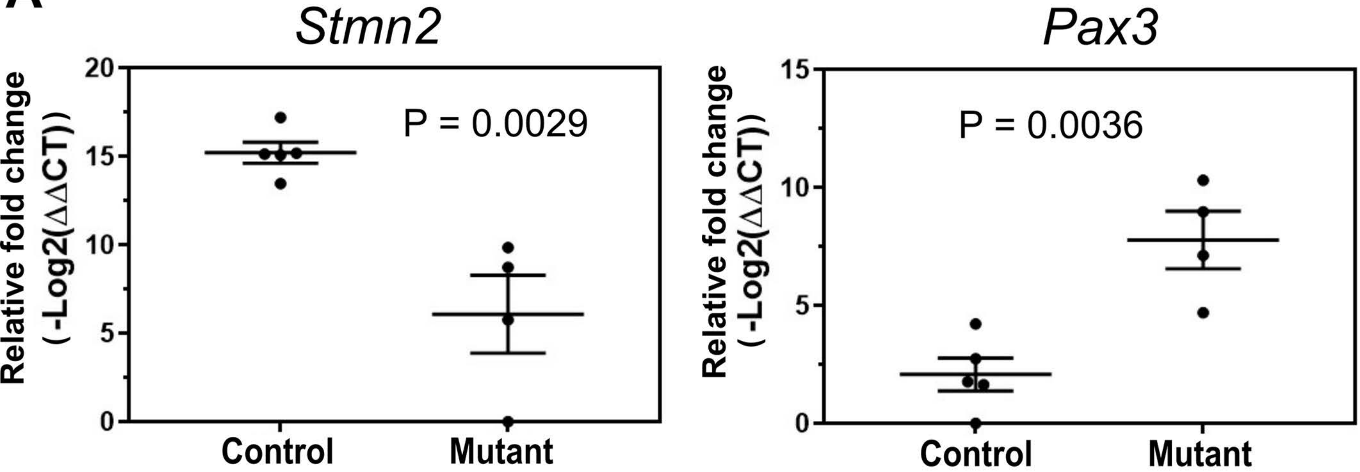

B

	Stmn2	*Pax3*
Log2 fold change using qRT-PCR	- 9.1	5.7
Log2 fold change using RNA-Seq	- 8.0	6.8

Control: *Wnt1Cre+; R26R-TdTomato+*

Mutant: *RarαDN$^{LoxP/+}$; Wnt1Cre+; R26R-TdTomato+*

Supplemental Figure 9

RNA-Seq correlates with immunohistochemistry

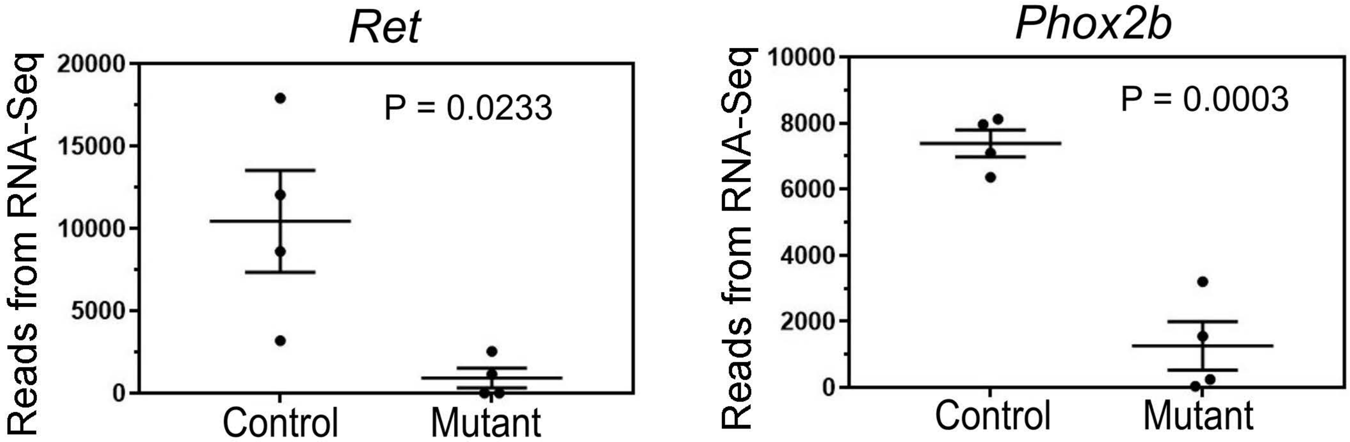

Control: *Wnt1Cre+; R26R-TdTomato+*

Mutant: *Rarα*$DN^{LoxP/+}$*; Wnt1Cre+; R26R-TdTomato+*
